# The effect of oral diabetes medications on glycated haemoglobin (HbA1c) in Asians in primary care: a retrospective cohort real-world data study

**DOI:** 10.1186/s12916-021-02221-z

**Published:** 2022-01-26

**Authors:** Hao Sen Andrew Fang, Qiao Gao, Wei Ying Tan, Mong Li Lee, Wynne Hsu, Ngiap Chuan Tan

**Affiliations:** 1 SingHealth Polyclinics, SingHealth, 167, Jalan Bukit Merah, Connection One, Tower 5, #15-10, Singapore, P.O. 150167 Singapore; 2 Institute of Data Science, National University of Singapore, Singapore, Singapore; 3 School of Computing, National University of Singapore, Singapore, Singapore; 4 Family Medicine Academic Clinical Programme, SingHealth-Duke NUS Academic Medical Centre, Singapore, Singapore

**Keywords:** Diabetes mellitus, Glycated hemoglobin, Antidiabetic agent, Asian, Primary care

## Abstract

**Background:**

Clinical trials have demonstrated that initiating oral anti-diabetic drugs (OADs) significantly reduce glycated hemoglobin (HbA1c) levels. However, variability in lifestyle modifications and OAD adherence impact on their actual effect on glycemic control. Furthermore, evidence on dose adjustments and discontinuation of OAD on HbA1c is lacking. This study aims to use real-world data to determine the effect of OAD initiation, up-titration, down-titration, and discontinuation on HbA1c levels, among Asian patients managed in primary care.

**Methods:**

A retrospective cohort study over a 5-year period, from Jan 2015 to Dec 2019 was conducted on a cohort of multi-ethnic adult Asian patients with clinical diagnosis of type 2 diabetes mellitus (T2DM) managed by a network of primary care clinics in Singapore. Nine OADs from five different classes (biguanides, sulphonyurea, dipeptidyl peptidase-4 [DPP-4] inhibitors, sodium-glucose cotransporter-2 [SGLT-2] inhibitors, and alpha-glucosidase inhibitors) were evaluated. Patients were grouped into “No OAD”, “Non-titrators,” and “Titrators” cohorts based on prescribing patterns. For the “Titrators” cohort, the various OAD titrations were identified. Subsequently, a descriptive analysis of HbA1c values before and after each titration was performed to compute a mean difference for each unique titration identified.

**Results:**

Among the cohort of 57,910 patients, 43,338 of them had at least one OAD titration, with a total of 76,990 pairs of HbA1c values associated with an OAD titration. There were a total of 206 unique OAD titrations. Overall, initiation of OADs resulted in a reduction of HbA1c by 3 to 12 mmol/mol (0.3 to 1.1%), respectively. These results were slightly lower than those reported in clinical trials of 6 to 14 mmol/mol (0.5 to 1.25%). The change of HbA1c levels due to up-titration, down-titration, and discontinuation were −1 to −8 mmol/mol (−0.1 to −0.7%), +1 to 7 mmol/mol (+0.1 to +0.6%), and +2 to 11 mmol/mol (+0.2 to +1.0%), respectively. The HbA1c lowering effect of initiating newer OADs, namely DPP-4 inhibitors and SGLT-2 inhibitors was 8 to 11 mmol/mol (0.7 to 0.9%) and 7 to 11 mmol/mol (0.6 to 1.0%), respectively.

**Conclusion:**

The real-world data on Asians with T2DM in this study show that the magnitudes of OAD initiation and dose titration are marginally lower than the results from clinical trials. During shared decision-making in selecting treatment options, the results enable physicians to communicate realistic expectation of the effect of oral medications on the glycemic control of their patients in primary care.

**Supplementary Information:**

The online version contains supplementary material available at 10.1186/s12916-021-02221-z.

## Background

The global diabetes prevalence was estimated to be 9.3% (463 million people) in 2019 and is expected to rise to 10.2% (578 million people) by 2030 [1]. Likewise, the global direct health expenditure of type 2 diabetes mellitus (T2DM) is projected to grow from USD 760 billion in 2019 to USD850 billion by 2030 [2]. To mitigate the rising health and economic burdens associated with T2DM, clinical guidelines advocate a multifaceted approach, including diet, lifestyle, and medications to achieve disease control, measured using glycated haemoglobin (HbA1c) [3–5].

Under the National Institute for Health and Care Excellence (NICE) guidelines, several classes of oral anti-diabetic drugs (OADs) are available for therapeutic treatment, including biguanides, sulphonyurea, dipeptidyl peptidase-4 (DPP-4) inhibitors, and sodium-glucose cotransporter-2 (SGLT-2) inhibitors [3]. While studies have evaluated the effectiveness of these OADs, most were based on Caucasian populations [6–8]. Asians have different vascular risk profiles from Caucasians [9, 10]. Literature suggests that due to pathophysiology, clinical presentation, and management, Asians suffer from higher propensity of developing T2DM and faster progression of the disease to complications [11–14]. Given that Asia is the epicenter of T2DM, accounting for 60% of the world’s population with T2DM, it thus becomes critical to understand the effectiveness of OADs in managing T2DM in Asians [15].

As most of the studies evaluating OAD effectiveness were conducted in controlled trial-based settings, it remains uncertain if the magnitude of HbA1c lowering differ in actual clinical practice due to suboptimal medication adherence, psychosocial profiles, lifestyle, and health-seeking behaviour [6–8]. Furthermore, data on OAD up-titration, down-titration, and discontinuation is currently lacking. T2DM is a chronic disease which often requires serial medication titrations to maintain glycemic control over the course of a person’s lifetime. This study aims to determine the HbA1c change following titration of various OADs from real-world data among Asians with T2DM managed in primary care. Understanding the impact of various OAD dosage titrations on HbA1c would enable physicians to provide clear expected outcomes of their recommended OAD changes to facilitate better shared, and more informed, decision-making with patients.

## Methods

### Study design, setting, and population

A retrospective cohort study was conducted using patient electronic medical records (EMR) from a network of eight polyclinics located in Singapore. These polyclinics manage about 1.8 million patient attendances annually and serves about 1.5 million multi-ethnic Asians (76.2% Chinese, 15.0% Malays, 7.4% Indians, 1.4% minority ethnic groups) living in the eastern region of Singapore [16]. About one third of patients who attend the polyclinics are aged 65 years and above.

Based on local clinical practice guidelines, patients with T2DM are reviewed by the physicians and nurse physicians with a HbA1c test performed at the in-house laboratory to assess their diabetic control once every 3 to 6 months, with flexibility for closer monitoring if their medical conditions are unstable [5]. Their demographic, clinical, and laboratory information are documented in the polyclinic EMR system.

The study population comprised multi-ethnic adult patients, aged 21 years or older, with diabetic-related diagnoses entered in the EMR (Table 1). Patients with type 1 diabetes were excluded. The clinical data of the study population were extracted from the EMR from January 1, 2015, to December 31, 2019.

**Table 1:** Diabetic-related diagnoses, with ICD-10 codes, used to identify eligible patients

Diabetic-related diagnosis	ICD-10 code
Type 2 diabetes without complication	E11.9
Type 2 diabetes with incipient diabetic nephropathy	E11.21
Type 2 diabetes with established diabetic nephropathy	E11.22
Type 2 diabetes with unspecified neuropathy	E11.40
Unspecified diabetes mellitus with background retinopathy	E14.31
Unspecified diabetes mellitus with foot ulcer due to multiple causes	E14.73

Diabetic-related diagnoses based on a restricted set of ICD-10 diagnosis codes used in the polyclinic electronic medical records system. Abbreviations: *ICD-10* International Classification of Diseases, 10th Revision

### Data definition and processing

Nine different types of OADs in the polyclinic drug formulary were examined for their clinical effectiveness. These OADS are metformin, glipizide, gliclazide, tolbutamide, sitagliptin, linagliptin, dapagliflozin, empagliflozin, and acarbose. The total daily dosage for each OAD was also computed. For example, if a patient was taking metformin 500mg twice daily, it would be converted to a dose of metformin 1000mg. In selected atypical cases when some patients were prescribed variable OAD doses across different days of the week, the mean daily dose over a week was used to compute the OAD dose.

Patients with fewer than two HbA1c values were excluded as at least two HbA1c values were needed for comparison of OAD effect. Patients who were not on OAD throughout the study period were grouped into the “No OAD” cohort. For the remaining patients, OAD titration was determined by analyzing their prescription records. The records were sorted by patient identifier and then by prescription date. Next, the time interval between two consecutive prescriptions for each patient were assessed for OAD discontinuation. A patient was considered to have discontinued taking OAD if there was no record of OAD within a year of the last prescription. The difference in OAD dose between two consecutive prescriptions indicate OAD dose titrations. Only pairs of consecutive prescriptions with dose titrations were selected for analysis. Patients without such pair of prescriptions were grouped into the “Non-titrators” cohort, while the rest were grouped into the “Titrators” cohort. Patients in these two cohorts could be on monotherapy or a combination of OADs or insulin. The different types of insulin (e.g., detemir, glargine) were combined into a single insulin group in the analysis.

The investigator first identified and collated all pairs of index prescriptions in the “Titrators’ cohort”. The unique sets of OAD titrations, which consisted of a corresponding pair of initial OAD dose and new OAD dose, were then extracted. An example of an OAD titration is metformin 500 to 1000mg. For each unique OAD titration, the *pre-HbA1c* indicated the latest glycemic control within 1 year before the OAD dose adjustment and the *post-HbA1c* reflected the glycemic control beyond 12 weeks to 1 year after the OAD titration. The HbA1c values measured within 12 weeks of titration of an OAD were excluded. This 12-week window was used as HbA1c values are derived from irreversible glucose binding on erythrocytes which have a lifespan of about 90 days, thus reflecting glycemic control over the past 3 months [17, 18]. It is also consistent with clinical trials for OAD typically have a minimum 12-week follow-up to assess HbA1c lowering efficacy [6]. The 1 year limitation concurred with the recommendation to perform panel tests annually for patients with T2DM based on local clinical practice guidelines [5].

**Fig. 1:**
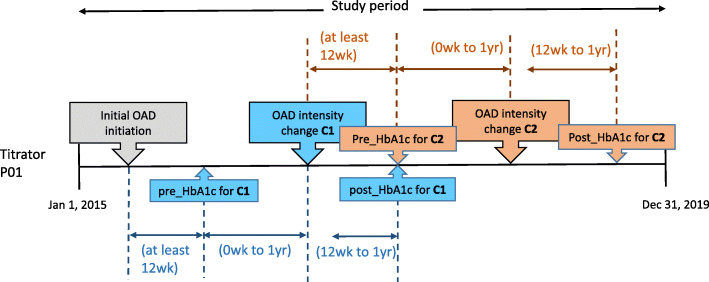
This figure illustrates the data processing to identify HbA1c pairs for patients in “Titrators” group. To obtain the HbA1c value before OAD titration (pre_HbA1c) and after OAD titration (post_HbA1c) for the analysis, an OAD titration is first identified. In the figure, two OAD titrations (C1 and C2) were identified for the patient (P01). For each OAD titration, the pre_HbA1c is taken to be the most recent HbA1c result within one year before an OAD titration, while the post_HbA1c is the first HbA1c value within twelve weeks to one year after the OAD titration. Abbreviations: C1 = first OAD titration, C2 = second OAD titration, P01 = illustrative patient, pre_HbA1c = HbA1c before OAD titration; post_HbA1c = HbA1c after the OAD titration

Since patients could have multiple OAD titrations, a HbA1c value could serve both as the *post-HbA1c* for one titration, as well as the *pre-HbA1c* for a subsequent titration (Fig. 1). In order to isolate the HbA1c change to each individual OAD titration, we excluded instances where there were multiple titrations (of the OAD of interest, other OADs or insulin) within the 1-year period after the OAD titration of interest.

For those in the “Non-titrators” and “No OAD” cohorts, each of their serial HbA1c values were collated and used as a comparison group. Similar to the “Titrators” cohort, pairs of consecutive HbA1c values with a minimum 12-week interval were used to correlate with the dose adjustment of the respective OAD.

Overall, the mean difference and 95% confidence interval (CI) between the *pre-HbA1c* and the *post-HbA1c* pairs were used to report the effect of OAD up-titration, down-titration, and no-titration. The descriptive analyses which included identifying the pairs, computing the difference in each *pre-HbA1c* and *post-HbA1c* pair and then aggregating the mean for each type of OAD titration was performed using the Python “statsmodel” package, version 0.12.1. To further investigate and adjust for the effect of covariates available in the dataset, a multivariate regression analysis was performed, using a multivariate linear regression model adjusting for covariates at each OAD titration. The adjusted change in HbA1c is the least-squares mean value of changes in HbA1c obtained from multivariable linear regression model. Missing data from biomarkers namely blood pressure readings (<1%), body mass index (10%), and lipid data (30%) were handled by imputing the population mean before fitting to the regression model. The rest of the covariates did not have missing values.

## Results

**Fig. 2:**
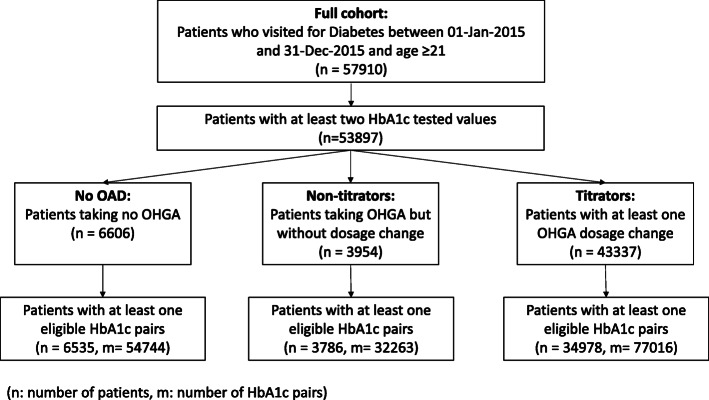
Flow chart illustrating the derivation of the patient cohorts. “No OAD” cohort refers to patients who were not on OAD throughout the study period. “Non-titrators” cohort refers to patients who were on at least one OAD but did not have any OAD dose adjustment during the study period. “Titrators” cohort refers to patients who were on at least one OAD and had at least one OAD dose adjustment during the study period. Abbreviation: n= number of patients, m = number of HbA1c pairs

A total of 57,910 unique adult patients with T2DM were extracted from the polyclinic EMR. 53,897 of them (93.1%) had at least two HbA1c values over the study period. Cohorts of 6535, 3784, and 34,978 patients in the “No OAD,” “Non-titrators,” and “Titrators” categories, respectively, were identified with at least one pre- and post-HbA1c pair. Analyses were conducted on 54,744, 32,262, and 77016 HbA1c pairs in the “No OAD,” “Non-titrators,” and “Titrators” cohorts respectively. Figure 2 shows the derivation of the three patient cohorts.

The baseline characteristics of the patients in each cohort are shown in Table 2. The mean age ranges from 64.0 to 71.9 years across the three cohorts, with slight female predominance (51.4–57.1%). The majority of patients in each cohort had Hypertension (87.5–91.0%) and Dyslipidemia (93.5–94.0%). In the “Titrators” group, most of the patients had T2DM for at least 3 years (53.8%).

**Table 2:** Baseline characteristics of patients in study cohort

Characteristics	No OAD (*n* = 6535)	Non-titrators (*n* = 3786)	Titrators (*n* = 34978)	Full cohort (*n* = 57910)
Total patients, *n* (%)	6535 (100)	3786 (100)	34978 (100)	57910 (100)
Age (year), mean (SD)	71.9 (11.0)	68.6 (10.9)	64.0 (10.9)	65.4 (11.8)
Sex, males, *n* (%)	2801 (42.9)	1788 (47.2)	16997 (48.6)	28107 (48.5)
Race, *n* (%)				
Chinese	5396 (82.6)	2876 (76.0)	24530 (70.1)	41036 (70.9)
Malay	600 (9.2)	471 (12.4)	5478 (15.7)	8741 (15.1)
Indian	339 (5.2)	284 (7.5)	3448 (9.9)	5525 (9.5)
Others	200 (3.1)	155 (4.1)	1549 (4.4)	2608 (4.5)
Body mass index (kg/m^2^), mean (SD)	25.4 (4.4)	25.9 (4.6)	26.7 (4.8)	26.5 (4.8)
Diagnosis, *n* (%)				
Dyslipidemia	6109(93.5)	3559(94.0)	32893(94.0)	53609(92.6)
Hypertension	5944(91.0)	3393(89.6)	30620(87.5)	50483(87.2)
Years with diabetes at base visit, *n* (%)				
0	5473 (83.7)	279 (7.4)	5190 (14.8)	14625 (25.3)
1	79 (1.2)	422 (11.1)	303 1(8.7)	4731 (8.2)
2	150 (2.3)	430 (11.4)	3120 (8.9)	4859 (8.4)
3	633 (9.7)	2494 (65.9)	22008 (62.9)	31137 (53.8)
4	63 (1.0)	53 (1.4)	448 (1.3)	715 (1.2)
≥5	137 (2.1)	108 (2.9)	1182 (3.4)	1843 (3.2)
Number of HbA1c tests per year, mean (SD)	2.1(0.9)	2.2(1.1)	3.2(0.9)	2.8(1.2)
Number of OAD prescribed, *n* (%)				
0	6535 (100.0)	9 (0.2)	3737 (10.7)	13176 (22.8)
1	0 (0.0)	2685 (70.9)	13234 (37.8)	20517 (35.4)
2	0 (0.0)	949 (25.1)	13741 (39.3)	18776 (32.4)
3	0 (0.0)	129 (3.4)	3745 (10.7)	4782 (8.3)
4	0 (0.0)	14 (0.4)	521 (1.5)	659 (1.1)
On insulin, *n* (%)	0 (0.0)	37 (1.0)	3868 (11.1)	5538 (9.6)
HbA1c group, *n* (%)				
<7.0	5735 (87.8)	2627 (69.4)	13886 (39.7)	26901 (46.5)
7.0–7.9	252 (3.9)	852 (22.5)	11425 (32.7)	15299 (26.4)
8.0–8.9	15 (0.2)	124 (3.3)	4292 (12.3)	5722 (9.9)
9.0–9.9	1 (0.0)	29 (0.8)	1771 (5.1)	2560 (4.4)
≥10	1 (0.0)	38 (1.0)	1828 (5.2)	2961 (5.1)
Missing^1^	531 (8.1)	116 (3.1)	1776 (5.1)	4467 (7.7)
Number of OAD prescriptions per year, mean (SD)	0.0 (0.0)	2.2 (1.3)	3.5 (1.2)	3.1 (1.5)

^1^These patients did not have a HbA1c test done at their baseline visit. Abbreviations: *HbA1c* glycated hemoglobin, *OAD* oral anti-diabetic drug, *SD* standard deviation

### Change in HbA1c following OAD titration

**Fig. 3:**
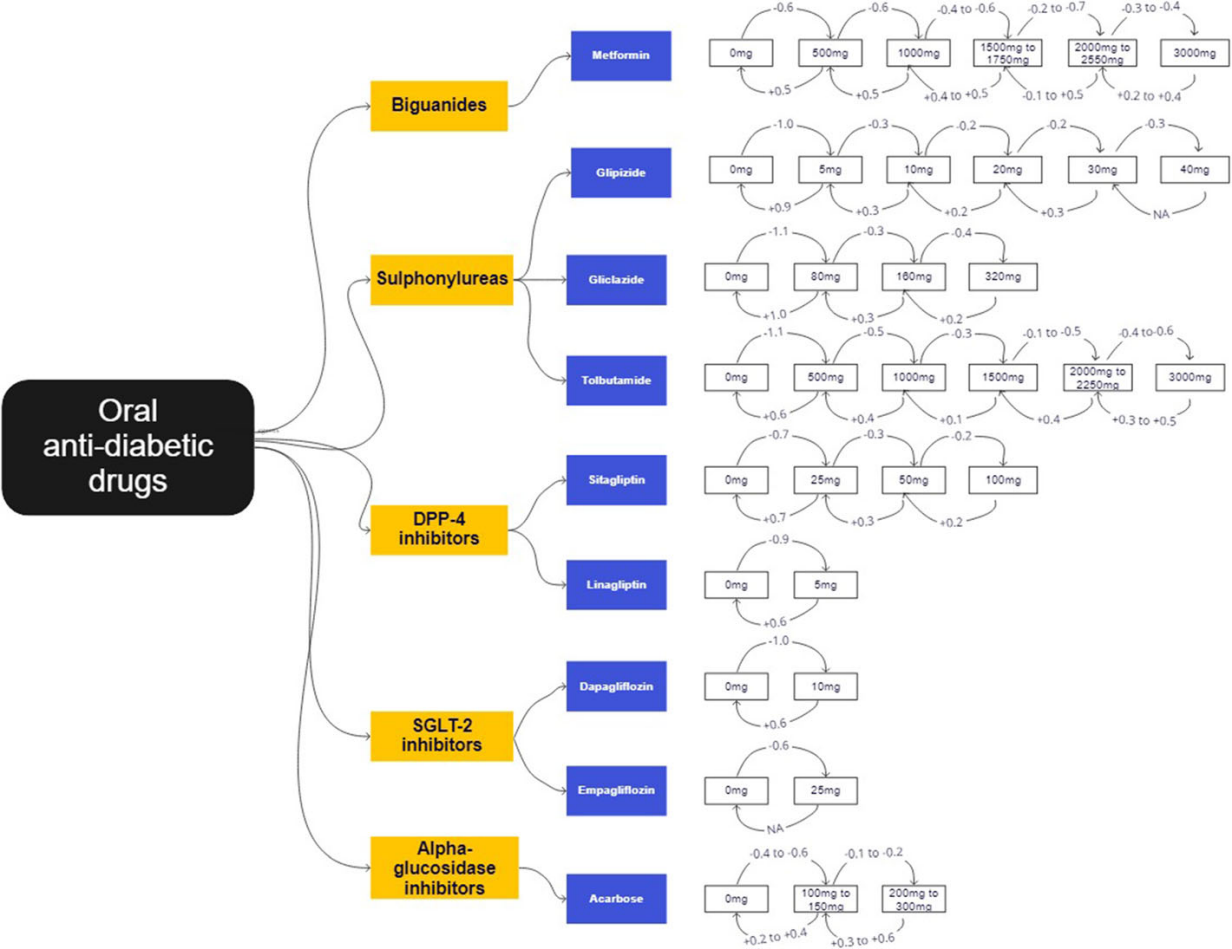
Executive summary of HbA1c change (with HbA1c denoted in mmol/mol) with various OAD titration. The dosages in the white rectangle boxes refer to the total daily dosage of the medication. The numbers on the arrows represent the HbA1c change. The direction of the arrows represents an up-titration (rightward arrow), or down-titration (leftward arrow). Abbreviations: HbA1c = glycated haemoglobin, OAD = oral anti-diabetic drug

There were a total of 206 unique sets of OAD dosage titrations, for the 9 different OADs—metformin (*n*=72), glipizide (*n*=41), gliclazide (*n*=22), tolbutamide (*n*=24), sitagliptin (*n*=12), linagliptin (*n*=6), dapagliflozin (*n*=8), empagliflozin (*n*=2), and acarbose (*n*=19). The mean difference in HbA1c for the various OAD titrations are shown in Tables 3, 4, 5, 6, 7, 8, 9, 10, 11. An executive summary of the results illustrating the more typical OAD titrations is found in Fig. 3. For those more familiar with HbA1c results in the National Glycohemoglobin Standardization Program (NGSP) network (represented in %), the same figure and tables with NGSP units can be found in the supplementary file as Additional file 1: Figure S1 and Additional file 1: Tables S1a to S1i.

**Table 3:** Change in HbA1c values (with HbA1c denoted in mmol/mol) after metformin titration

Metformin dose after titration												
0	125	250	500	750	850	1000	1500	1700	2000	2250	2550	3000
		-3(-4, -3)#m=487	-7(-7, -7)#m=1849			-10(-11, -9)#m=676	-8(-12, -3)#m=36	-7(-11, -3)#m=60				
		-4(-7, -2)#m=34										
6(4, 8)#m=173	2(1, 3)#m=105		-4(-6, -4)#m=906			-10(-13, -8)#m=87						
6(4, 8)#m=220		3(3, 4)#m=1412		-3(-4, -2)#m=282		-7(-7, -7)#m=3108	-9(-12, -6)#m=77	-14(-18, -11)#m=53				
			2(1, 3)#m=182			-3(-4, -2)#m=225	-7(-9, -6)#m=190	-8(-12, -4)#m=31				
			3(-0, 6)#m=34			1(-1, 3)#m=58		-3(-6, -2)#m=161			-2(-6, 0)#m=39	
8(4, 10)#m=137		8(3, 12)#m=31	6(4, 6)#m=1953	-1(-2, 0)#m=105	-1(-3, 0)#m=57		-4(-6, -5)#m=2150	-7(-7, -6)#m=1670	-4(-7, -3)#m=198		-10(-14, -7)#m=47	-6(-8, -2)#m=47
12(7, 16)#m=41			8(5, 11)#m=96	3(1, 6)#m=91		4(3, 4)#m=1128		-3(-3, -2)#m=739	-4(-6, -3)#m=418	-7(-7, 6)#m=498	-8(-9, -7)#m=398	
13(8, 19)#m=46			8(3, 12)#m=38		2(1, 3)#m=240	6(6, 7)#m=773	1(0, 2)#m=142		-2(-3, -2)#m=611		-6(-6, -4)#m=1154	
						4(3, 6)#m=228	3(1, 6)#m=82	3(1, 4)#m=127			-3(-4, -2)#m=179	-3(-6, -2)#m=458
						6(2, 9)#m=34	3(3, 4)#m=227	1(-2, 3)#m=30	1(-2, 3)#m=42		-4(-4, -3)#m=311	-4(-7, -2)#m=124
					3(0, 7)#m=30	6(3, 9)#m=75	6(4, 7)#m=165	3(3, 4)#m=920	-1(-2, 0)#m=182	3(-0, 6)#m=33		-3(-4, -2)#m=642
						10(6, 14)#m=41	9(6, 11)#m=65		4(3, 4)#m=438	4(2, 7)#m=35	2(0, 3)#m=129	

Change in HbA1c values after metformin initiation, titration, or discontinuation. The values above the diagonal represent the instances where the medication has been initiated or up-titrated, while the values below the diagonal represent instances where the medication has been down-titrated or discontinued. The values refer to the mean difference in HbA1c (MD) and 95% confidence intervals. MD below 0 indicate a lowering in HbA1c while those above 0 indicate an increase in HbA1c. #m refers to the number of HbA1c pairs for that dose titration

**Table 4:** Change in HbA1c values (with HbA1c denoted in mmol/mol) after glipizide titration

Glipizide dose after titration										
0	2.5	5	7.5	10	12.5	15	20	25	30	40
	-8(-9, -7)#m=471	-11(-12, -10)#m=1283		-13(-14, -11)#m=364			-1(-7, 3)#m=41			
8(8, 9)#m=596		-6(-6, -4)#m=660		-9(-14, -3)#m=51						
10(10, 11)#m=818	4(3, 4)#m=815		-4(-7, -1)#m=82	-3(-3, -2)#m=1686						
		2(0, 6)#m=59		-0(-2, 1)#m=157		-0(-1, 1)#m=133				
10(9, 12)#m=260	3(1, 6)#m=32	3(3, 3)#m=1042	0(-1, 1)#m=267			-2(-2, -1)#m=966	-2(-3, -2)#m=753			
								0(-2, 2)#m=57		
		1(-1, 3)#m=30	1(0, 3)#m=50	2(1, 3)#m=376			-1(-1, 0)#m=852		-1(-2, 1)#m=294	
10(6, 13)#m=47				2(2, 3)#m=514	-1(-2, 0)#m=145	0(0, 1)#m=362		-2(-3, -1)#m=617	-1(-2, 0)#m=465	
						0(-2, 2)#m=72	1(-1, 2)#m=95		-1(-2, 0)#m=444	
7(2, 10)#m=45				2(-1, 4)#m=68		3(2, 4)#m=259	3(2, 4)#m=323	2(0, 3)#m=83		-3(-6, 0)#m=73

Change in HbA1c values after glipizide initiation, titration or discontinuation. The values above the diagonal represent the instances where the medication has been initiated or up-titrated, while the values below the diagonal represent instances where the medication has been down-titrated or discontinued. The values refer to the mean difference in HbA1c (MD) and 95% confidence intervals. MD below 0 indicate a lowering in HbA1c while those above 0 indicate an increase in HbA1c. #m refers to the number of HbA1c pairs for that dose titration

**Table 5:** Change in HbA1c values (with HbA1c denoted in mmol/mol) after gliclazide titration

Gliclazide dose after titration									
0	30	40	60	80	90	120	160	240	320
	-10(-13, -8)#m=57	-12(-14, -10)#m=89		-12(-14, -11)#m=174					
8(4, 10)#m=44			-2(-4, 0)#m=73						
13(10, 15)#m=108				-6(-7, -3)#m=109					
	4(2, 6)#m=49				-3(-7, 1)#m=38	-1(-6, 3)#m=33			
11(9, 13)#m=91		3(2, 4)#m=171					-3(-4, -2)#m=233		
								-3(-6, -1)#m=34	
				3(2, 4)#m=172		-1(-4, 1)#m=59		-3(-6, -1)#m=113	-4(-7, -2)#m=112
							1(-2, 3)#m=50		-2(-4, 1)#m=77
							2(1, 4)#m=119	3(-2, 8)#m=31	

Change in HbA1c values after gliclazide initiation, titration, or discontinuation. The values above the diagonal represent the instances where the medication has been initiated or up-titrated, while the values below the diagonal represent instances where the medication has been down-titrated or discontinued. The values refer to the mean difference in HbA1c (MD) and 95% confidence intervals. MD below 0 indicate a lowering in HbA1c while those above 0 indicate an increase in HbA1c. #m refers to the number of HbA1c pairs for that dose titration

**Table 6:** Change in HbA1c values (with HbA1c denoted in mmol/mol) after tolbutamide titration

Tolbutamide dose after titration								
0	250	500	750	1000	1500	2000	2250	3000
	-8(-11, -6)#m=52	-12(-14, -9)#m=103						
6(3, 7)#m=90		-6(-8, -3)#m=92						
7(6, 8)#m=177	3(2, 4)#m=243		-3(-7, 0)#m=44	-6(-7, -4)#m=203				
		1(-1, 2)#m=89		1(-3, 6)#m=37	-6(-8, -2)#m=52			
8(3, 11)#m=45		4(3, 4)#m=277			-3(-6, -2)#m=187			
			3(2, 6)#m=64	1(0, 2)#m=190		-1(-4, 2)#m=42	-6(-8, -3)#m=96	
				7(3, 11)#m=50				-7(-11, -3)#m=42
					4(3, 6)#m=95			-4(-7, -2)#m=62
					6(2, 9)#m=43	3(2, 6)#m=67		

Change in HbA1c values after tolbutamide initiation, titration, or discontinuation. The values above the diagonal represent the instances where the medication has been initiated or up-titrated, while the values below the diagonal represent instances where the medication has been down-titrated or discontinued. The values refer to the mean difference in HbA1c (MD) and 95% confidence intervals. MD below 0 indicate a lowering in HbA1c while those above 0 indicate an increase in HbA1c. #m refers to the number of HbA1c pairs for that dose titration

**Table 7:** Change in HbA1c values (with HbA1c denoted in mmol/mol) after sitagliptin titration

Sitagliptin dose after titration				
0	25	50	75	100
	-8(-9, -6)#m=266	-8(-9, -7)#m=411		-6(-9, -2)#m=86
8(6, 9)#m=93		-3(-4, -2)#m=225		
8(6, 10)#m=156	3(0, 6)#m=56		-3(-7, 0)#m=43	-2(-4, -1)#m=439
				1(-3, 6)#m=35
7(4, 9)#m=82		2(0, 3)#m=91		

Change in HbA1c values after sitagliptin initiation, titration, or discontinuation. The values above the diagonal represent the instances where the medication has been initiated or up-titrated, while the values below the diagonal represent instances where the medication has been down-titrated or discontinued. The values refer to the mean difference in HbA1c (MD) and 95% confidence intervals. MD below 0 indicate a lowering in HbA1c while those above 0 indicate an increase in HbA1c. #m refers to the number of HbA1c pairs for that dose titration

**Table 8:** Change in HbA1c values (with HbA1c denoted in mmol/mol) after linaliptin titration

Linagliptin dose after titration		
0	2.5	5
	-9(-11, -7)#m=139	-10(-10, -9)#m=1807
6(2, 9)#m=32		-3(-6, -1)#m=79
7(6, 8)#m=363	2(0, 4)#m=36	

Change in HbA1c values after linagliptin initiation, titration, or discontinuation. The values above the diagonal represent the instances where the medication has been initiated or up-titrated, while the values below the diagonal represent instances where the medication has been down-titrated or discontinued. The values refer to the mean difference in HbA1c (MD) and 95% confidence intervals. MD below 0 indicate a lowering in HbA1c while those above 0 indicate an increase in HbA1c. #m refers to the number of HbA1c pairs for that dose titration

**Table 9:** Change in HbA1c values (with HbA1c denoted in mmol/mol) after dapagliflozin titration

Dapagliflozin dose after titration			
0	2.5	5	10
	-9(-11, -6)#m=67	-9(-10, -8)#m=570	-11(-11, -10)#m=722
		-1(-4, 2)#m=39	
3(1, 7)#m=85			-3(-4, -2)#m=264
7(3, 9)#m=96		1(-1, 3)#m=76	

Change in HbA1c values after dapagliflozin initiation, titration, or discontinuation. The values above the diagonal represent the instances where the medication has been initiated or up-titrated, while the values below the diagonal represent instances where the medication has been down-titrated or discontinued. The values refer to the mean difference in HbA1c (MD) and 95% confidence intervals. MD below 0 indicate a lowering in HbA1c while those above 0 indicate an increase in HbA1c. #m refers to the number of HbA1c pairs for that dose titration

**Table 10:** Change in HbA1c values (with HbA1c denoted in mmol/mol) after empagliflozin titration

Empagliflozin dose after titration		
0	12.5	25
	-8(-10, -6)#m=152	-7(-9, -5)#m=101

Change in HbA1c values after empagliflozin initiation, titration, or discontinuation. The values above the diagonal represent the instances where the medication has been initiated or up-titrated, while the values below the diagonal represent instances where the medication has been down-titrated or discontinued. The values refer to the mean difference in HbA1c (MD) and 95% confidence intervals. MD below 0 indicate a lowering in HbA1c while those above 0 indicate an increase in HbA1c. #m refers to the number of HbA1c pairs for that dose titration

**Table 11:** Change in HbA1c values (with HbA1c denoted in mmol/mol) after acarbose titration

Acarbose dose after titration					
0	50	100	150	200	300
	-3(-6, -1)#m=49	-4(-7, -3)#m=221	-7(-8, -4)#m=211		-7(-11, -1)#m=37
2(0, 4)#m=52		-3(-8, 0)#m=34			
4(2, 6)#m=149	3(1, 6)#m=40		-2(-6, 0)#m=72	-2(-4, 0)#m=94	
4(2, 6)#m=122		0(-1, 2)#m=65			-2(-3, 0)#m=144
4(2, 7)#m=67		3(2, 6)#m=48			-1(-3, 1)#m=75
7(4, 10)#m=82			7(3, 9)#m=49	3(2, 6)#m=92	

Change in HbA1c values after acarbose initiation, titration, or discontinuation. The values above the diagonal represent the instances where the medication has been initiated or up-titrated, while the values below the diagonal represent instances where the medication has been down-titrated or discontinued. The values refer to the mean difference in HbA1c (MD) and 95% confidence intervals. MD below 0 indicate a lowering in HbA1c while those above 0 indicate an increase in HbA1c. #m refers to the number of HbA1c pairs for that dose titration

Initiation of OADs resulted in a lowering of HbA1c by 3 to 12 mmol/mol (0.3 to 1.1%). Among the instances of OAD initiation, most commonly started OAD dosages were metformin 500mg (*m*=1849), glipizide 5mg (*m*=1283), and linagliptin (*m*=1807).

Up-titration of OAD resulted in a mean difference of −1 to −8 mmol/mol (−0.1 to −0.7%) in HbA1c, while down-titration resulted in a mean difference of +1 to +7 mmol/mol (+0.1 to +0.6%). Discontinuation of OADs resulted in an increase of HbA1c by 2 to 11 mmol/mol (+0.2 to +1.0%).

Among the various OADs, metformin had the largest overall cumulative HbA1c reduction from initiation to maximum dose −23 to −32 mmol/mol (−2.1 to −2.9%). The sulphonylurea group (glipizide, gliclazide, and tolbutamide) had the largest HbA1c reduction on initiation of 11 to 12 mmol/mol (−1.0 to −1.1%). The newer OAD groups such as DPP-4 inhibitors and SGLT-2 inhibitors, represented by sitagliptin and linagliptin; and dapagliflozin and empagliflozin, respectively, had a more modest HbA1c lowering effect on initiation of 8 to 10 mmol/mol (0.7 to 0.9%) and 7 to 11 mmol/mol (0.6 to 1.0%), respectively. Acarbose had the lowest HbA1c reduction on initiation of 4 to 7 mmol/mol (0.4 to 0.6%) and on up-titration of 1 to 2 mmol/mol (0.1 to 0.2%).

For comparison, among the HbA1c pairs from patients in the “No OAD” and “Non-titrators” cohort, no significant HbA1c change was noted in these two groups. Detailed results from this analysis can be found in the Additional file 2: Table S1. Multivariate regression to adjust for covariates which include gender, race, number of OADs prescribed, insulin use, and time to follow-up revealed that baseline HbA1c and insulin use had the largest impact of 2 to 4 mmol/mol (0.2 to 0.4%). A visualization of the covariate weights and the adjusted results can be found in the Additional file 2: Fig S1, Additional file 2: Table S2 and Additional file 2: Tables S3a to S3i.

## Discussion

The study enhances our understanding of the effect size resultant from various OAD dose titrations on HbA1c beyond trial-based studies. The latter tends to focus on regimental dose initiations or up-titrations [6–8]. Furthermore, findings from our study concur with the systematic review, which reports that OAD up-titration results in lower effect on HbA1c compared to OAD initiation [6]. In this study, any OAD initiation for an Asian patient with T2DM does not result in the expected magnitude of HbA1c reduction, being 3 to 12 mmol/mol (0.3 to 1.1%) lower compared to results from clinical trials [19–21]. A review of clinical trials published by Sherifali et al. found that most OADs lowered HbA1c levels by 6 to 14 mmol/mol (0.5 to 1.25%) [6]. One possible reason for this discrepancy is due to suboptimal medication adherence among patients in the real world [22]. This is supported by the fact that newer single daily dose of OADs (DPP-4 inhibitors and SGLT-2 inhibitors) achieve comparable HbA1c reduction with those from clinical trials [23–25]. The simplified dosing of newer OADs is postulated to result in better medication adherence and maintain its effectiveness in real-world setting vis-à-vis to clinical trials [26]. In contrast, patients taking multiple doses of metformin and sulphonylurea are often associated with poorer medication adherence and consequently attain lower HbA1c decline than expected [19, 20].

The OAD dose up-titration effect on HbA1c reduction of 1 to 8 mmol/mol (0.1 to 0.7%) in this study is comparable to the 2.6mmol/mol (0.2%) HbA1c decline after OAD dose escalation in an American-based real-world study [27]. Genetic variations between Caucasians and Asians and the resultant differences in pharmacokinetic and pharmacodynamics effects of OAD could account for the difference [28]. A trial comparing linagliptin 5mg dose among Japanese, Asian, and Caucasian patients with T2DM also revealed greater HbA1c reduction in the Japanese and Asian (non-Japanese) groups relative to those in the Caucasians [29].

The results reveal that cumulative HbA1c reduction is greater with incremental dose up-titrations rather than a large increase in dose. For example, a dose increase from metformin 0 to 1000mg resulted in a 0.9% reduction in HbA1c, while a phased increase from 0 to 500mg, and then from 500 to 1000mg would have resulted in a 13 mmol/mol (1.2%) reduction in HbA1c. Patients who are started on higher doses of medications may have lower medication adherence due to concern of adverse effects with higher doses, such as hypoglycemia [30]. Hence, a phased approach to medication up-titration is not only prudent to minimize adverse effects, but also more effective in achieving improved glycemic control.

### Study strength and limitations

Analysis of real-world data of a captive population of Asian patients constitutes a strength in this study. This allows us to account for the effects of real-world practicalities such as suboptimal medication adherence and may be more generalizable to patients in everyday practice. Such real-world evidence have become increasingly important in providing evidence for treatment effectiveness in clinical practice and can complement results from clinical trials in setting more realistic expectations for both physicians and patients on the attainment of HbA1c treatment goals [31].

Another benefit of using real-world data is the opportunity to gain insight on the impact of down-titrating and discontinuing OADs on HbA1c levels. Such changes would be challenging to elucidate from clinical trials due to protocol design and implementation reasons. Clinical practice is ideally centered on physician-patient shared decision-making in the selection of pharmacotherapy and addressing concerns of treatment options. Intentional dose adjustment is applied on the emergence of known side-effects from the medication. Including allergies and hypoglycemia from OAD. However, unilateral and undisclosed medication dose adjustments seem to be common in clinical practice secondary to a variety of reasons, from patients’ perceived adverse effects, related or unrelated to the OAD, to their own volition or ideation without physician input. Such behavior appears prevalent from our study data, with 16,174 instances of OAD down-titration and 4306 instances of OAD discontinuation. These results provide an estimation of magnitude change in glycemic control in the subset of the patients with reduction or discontinuity of their OAD therapy. Nevertheless, the magnitude on HbA1c increase in this study was lower for OAD down-titration and discontinuation than its dose escalation. This may be an avenue for further research into a possible sustained effect of OAD on HbA1c even after dose-reduction or discontinuation.

This study has its limitations. It uses an observational and retrospective study design and cannot account for unobserved heterogeneity (e.g., in diet and exercise, major health events) as would be in a prospective clinical trial. Nevertheless, until a large-scale prospective trial involving OADs an Asian primary care patients, this study will provide the information on the real-world effectiveness of a variety of OADs in this population. Furthermore, such study designs have also been used to generate valuable insights into OAD effectiveness on other populations [32–34]. Another limitation is that various time intervals between OAD titration and HbA1c tests were used (more than 12 weeks for pre-HbA1c and 12 weeks to 1 year for post-HbA1c). However, this is mitigated by evidence that changes in HbA1c after OAD titration tend to plateau after 12 weeks [35]. While recognizing that the HbA1c lowering effect was lesser in than found in trial-based studies, the latter were centered largely on Caucasian populations. Therefore, the effect sizes of OADs on glycemic control may not be applicable to non-Asians, considering the genetic differences in OAD pharmacology. This study covered only the OADs available in the drug formulary of the institution. Other classes of OAD such as the meglitinide and thiazolidinedione were excluded. The concomitant decreased drug clearance from renal impairment in chronic kidney diseases, drug-drug interactions, and the use of insulin in combination with OADs were not examined on their impact on the results. In order to mitigate some of these limitations, cases with concurrent OAD titrations were also excluded, such that the only difference between the pre-titration and post-titration HbA1c was related to a single OAD dose titration.

Beyond clinical outcome focusing on glycemic control, resources required to deliberate OAD dose titration including additional time required in the shared decision-making process and the training of the physicians to competently address the concerns need to be examined in future research. The results from this study are collated and condensed for easy reference in Fig. 3. We envision that physicians would refer to this executive summary in clinical practice as they propose OAD initiation and titration to patients with the goal of achieving HbA1c targets. Compared to trial-based studies, the expected outcomes from real-world experience may be more relatable to everyday patients. Going further, this method of using real-world data to generate real-world evidence may be applied in other settings to cost-effectively provide localized medication treatment effectiveness information. In the meantime, the investigators are currently developing an artificial intelligence-based counseling tool that will incorporate the study results to better empower both patients and physicians with information to decide on their preferred OAD therapy, so as to achieve optimal clinical outcomes, minimal adverse effects, and best possible patient-centered care.

## Conclusions

This study provides real-world evidence to elucidate the effect of OAD dose titration on HbA1c levels in Asian patients with T2DM. The real-world data in primary care showed lower HbA1c reduction after OAD dose escalation compared to results reported in clinical trials. In addition, this study also provides insights into OAD down-titration and discontinuation, which results in lower impact on HbA1c level than its corresponding up-titration or initiation. In clinical practice, these findings set realistic expectations for physicians to achieve the glycemic control of their patients during OAD dose titrations.

## Supplementary Information

**Additional file 1.** Additional file 1 of mean difference in HbA1c for the various OAD titrations and an executive summary denoted in % units. Figure S1 and Tables S1a to S1i. Figure S1. An executive summary of the results illustrating typical OAD titrations (with HbA1c denoted in %). Tables S1a to S1i. Changes in HbA1c values (with HbA1c denoted in %) after metformin titration.

**Additional file 2.** Additional file 2 of results from the analysis of “No OAD” and “Non-titrator” cohorts and multivariate analysis. Table S1, Figure S1, Table S2 and Table S3. Table S1. Change in HbA1c values (with HbA1c denoted in mmol/mol) in “No OAD” and “Non-titrators” cohorts. Figure S1. Visualization of covariates adjusted for in multivariate regression. Table S2. Multiple regression covariate weights (with HbA1c denoted in mmol/mol). Tables S3a to S3i. Change in HbA1c values (with HbA1c denoted in mmol/mol) after metformin titration (adjusted by regression).

## Data Availability

The datasets analyzed during the current study are not publicly available as they contain information that are sensitive to the study institution. They may be made available from the corresponding author on reasonable request.

## Abbreviations

DPP-4 inhibitor: Dipeptidyl peptidase-4 inhibitor
EMR: Electronic medical records
HbA1c: Glycated haemoglobin
ICD-10: International Classification of Diseases, 10^th^ Revision
NGSP: National Glycohemoglobin Standardization Program
NICE: National Institute for Health and Care Excellence
OAD: Oral anti-diabetic drug
SGLT-2 inhibitor: Sodium-glucose cotransporter-2 inhibitor
T2DM: Type-2 diabetes mellitus
