# Supplementary material for: The effect of oral diabetes medications on glycated haemoglobin (HbA1c) in Asians in primary care: a retrospective cohort real-world data study
Source: BMC Med. 2022 Jan 26;20:22. doi: 10.1186/s12916-021-02221-z (PMC8790837; doi:10.1186/s12916-021-02221-z)
Supplement: Supplementary file 1 — Additional file 1. Additional file 1 of mean difference in HbA1c for the various OAD titrations and an executive summary denoted in % units. Figure S1 and Tables S1a to S1i. Figure S1. An executive summary of the results illustrating typical OAD titrations (with HbA1c denoted in %). Tables S1a to S1i. Changes in HbA1c values (with HbA1c denoted in %) after metformin titration. [file 12916_2021_2221_MOESM1_ESM.docx]

Figure S1.


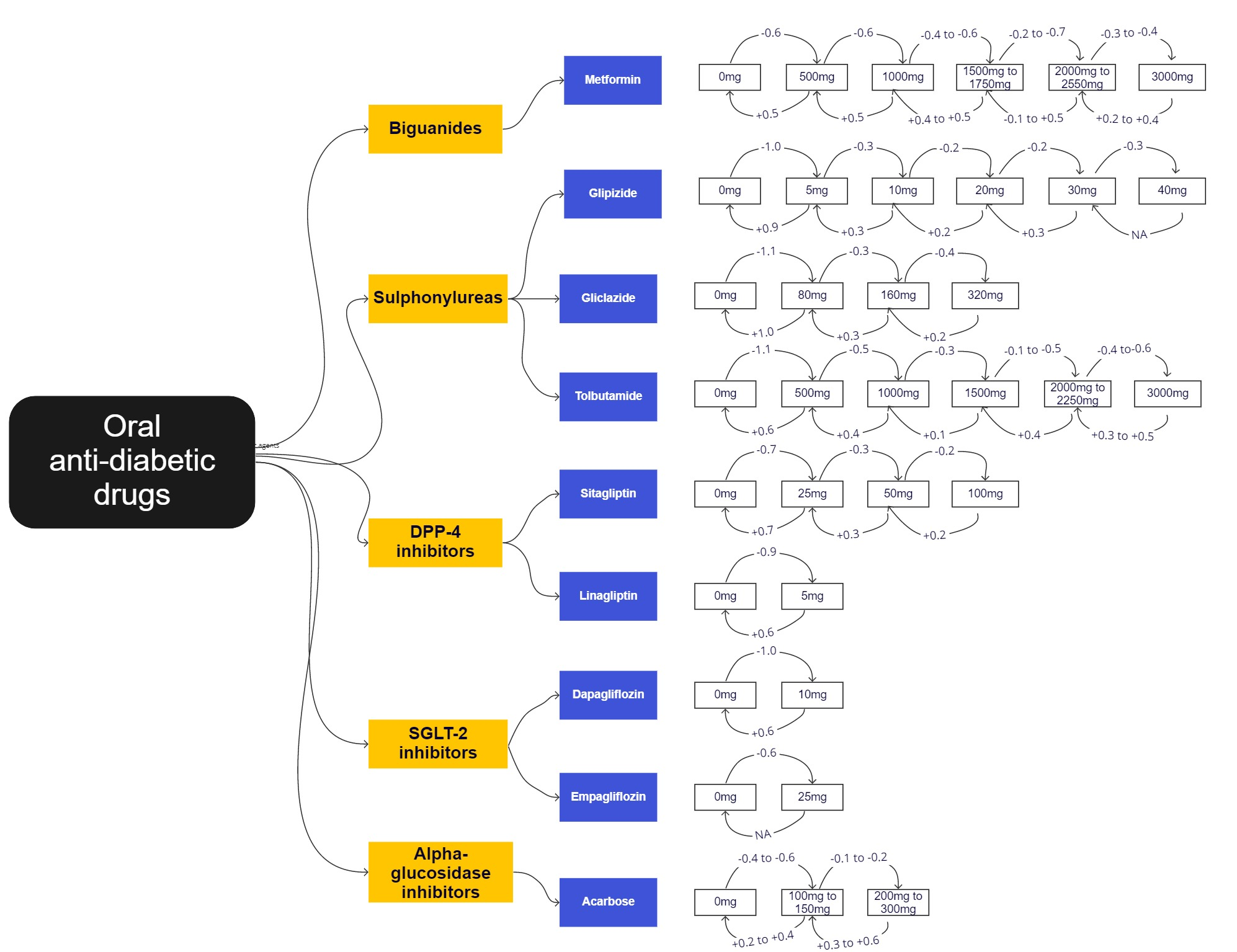
*Executive summary of HbA1c change (with HbA1c denoted in % units) with various OAD titration. The dosages in the white rectangle boxes refer to the total daily dosage of the medication. The numbers on the arrows represent the HbA1c change. The direction of the arrows represents an up-titration (rightward arrow), or down-titration (leftward arrow). Abbreviations: HbA1c = glycated haemoglobin, OAD = oral anti-diabetic drug.*

Table S1a. Change in HbA1c values (with HbA1c denoted in % units) after metformin titration.

|  |  | **Metformin dose after titration** | | | | | | | | | | | | |
| --- | --- | --- | --- | --- | --- | --- | --- | --- | --- | --- | --- | --- | --- | --- |
|  |  | **0** | **125** | **250** | **500** | **750** | **850** | **1000** | **1500** | **1700** | **2000** | **2250** | **2550** | **3000** |
| **Metformin dose before titration** | **0** |  |  | -0.3 (-0.4, -0.3) #m=487 | -0.6 (-0.6, -0.6) #m=1849 |  |  | -0.9 (-1.0, -0.8) #m=676 | -0.7 (-1.1, -0.3) #m=36 | -0.6 (-1.0, -0.3) #m=60 |  |  |  |  |
|  | **125** |  |  | -0.4 (-0.6, -0.2) #m=34 |  |  |  |  |  |  |  |  |  |  |
|  | **250** | 0.5 (0.4, 0.7) #m=173 | 0.2 (0.1, 0.3) #m=105 |  | -0.4 (-0.5, -0.4) #m=906 |  |  | -0.9 (-1.2, -0.7) #m=87 |  |  |  |  |  |  |
|  | **500** | 0.5 (0.3, 0.7) #m=220 |  | 0.3 (0.3, 0.4) #m=1412 |  | -0.3 (-0.4, -0.2) #m=282 |  | -0.6 (-0.6, -0.6) #m=3108 | -0.8 (-1.1, -0.5) #m=77 | -1.3 (-1.6, -1.0) #m=53 |  |  |  |  |
|  | **750** |  |  |  | 0.2 (0.1, 0.3) #m=182 |  |  | -0.3 (-0.4, -0.2) #m=225 | -0.6 (-0.8, -0.5) #m=190 | -0.7 (-1.1, -0.4) #m=31 |  |  |  |  |
|  | **850** |  |  |  | 0.3 (-0.0, 0.5) #m=34 |  |  | 0.1 (-0.1, 0.3) #m=58 |  | -0.3 (-0.5, -0.2) #m=161 |  |  | -0.2 (-0.5, 0.0) #m=39 |  |
|  | **1000** | 0.7 (0.4, 0.9) #m=137 |  | 0.7 (0.3, 1.1) #m=31 | 0.5 (0.4, 0.5) #m=1953 | -0.1 (-0.2, 0.0) #m=105 | -0.1 (-0.3, 0.0) #m=57 |  | -0.4 (-0.5, -0.4) #m=2150 | -0.6 (-0.6, -0.5) #m=1670 | -0.4 (-0.6, -0.3) #m=198 |  | -0.9 (-1.3, -0.6) #m=47 | -0.5 (-0.7, -0.2) #m=47 |
|  | **1500** | 1.1 (0.6, 1.5) #m=41 |  |  | 0.7 (0.4, 1.0) #m=96 | 0.3 (0.1, 0.5) #m=91 |  | 0.4 (0.3, 0.4) #m=1128 |  | -0.3 (-0.3, -0.2) #m=739 | -0.4 (-0.5, -0.3) #m=418 | -0.6 (-0.6, -0.5) #m=498 | -0.7 (-0.8, -0.6) #m=398 |  |
|  | **1700** | 1.2 (0.7, 1.7) #m=46 |  |  | 0.7 (0.3, 1.1) #m=38 |  | 0.2 (0.1, 0.3) #m=240 | 0.5 (0.5, 0.6) #m=773 | 0.1 (0.0, 0.2) #m=142 |  | -0.2 (-0.3, -0.2) #m=611 |  | -0.5 (-0.5, -0.4) #m=1154 |  |
|  | **2000** |  |  |  |  |  |  | 0.4 (0.3, 0.5) #m=228 | 0.3 (0.1, 0.5) #m=82 | 0.3 (0.1, 0.4) #m=127 |  |  | -0.3 (-0.4, -0.2) #m=178 | -0.3 (-0.5, -0.2) #m=458 |
|  | **2250** |  |  |  |  |  |  | 0.5 (0.2, 0.8) #m=34 | 0.3 (0.3, 0.4) #m=227 | 0.1 (-0.2, 0.3) #m=30 | 0.1 (-0.2, 0.3) #m=42 |  | -0.4 (-0.4, -0.3) #m=311 | -0.4 (-0.6, -0.2) #m=124 |
|  | **2550** |  |  |  |  |  | 0.3 (0.0, 0.6) #m=30 | 0.5 (0.3, 0.8) #m=75 | 0.5 (0.4, 0.6) #m=165 | 0.3 (0.3, 0.4) #m=920 | -0.1 (-0.2, 0.0) #m=182 | 0.3 (-0.0, 0.5) #m=33 |  | -0.3 (-0.4, -0.2) #m=642 |
|  | **3000** |  |  |  |  |  |  | 0.9 (0.5, 1.3) #m=41 | 0.8 (0.5, 1.0) #m=65 |  | 0.4 (0.3, 0.4) #m=438 | 0.4 (0.2, 0.6) #m=35 | 0.2 (0.0, 0.3) #m=129 |  |

Change in HbA1c values after metformin initiation, titration or discontinuation. The values above the diagonal represent the instances where the medication has been initiated or up-titrated, while the values below the diagonal represent instances where the medication has been down-titrated or discontinued. The values refer to the mean difference in HbA1c (MD) and 95% confidence intervals. MD below 0 indicate a lowering in HbA1c while those above 0 indicate an increase in HbA1c. #m refers to the number of HbA1c pairs for that dose titration.

Table S1b. Change in HbA1c values (with HbA1c denoted in % units) after glipizide titration.

|  |  | **Glipizide dose after titration** | | | | | | | | | | |
| --- | --- | --- | --- | --- | --- | --- | --- | --- | --- | --- | --- | --- |
|  |  | **0** | **2.5** | **5** | **7.5** | **10** | **12.5** | **15** | **20** | **25** | **30** | **40** |
| **Glipizide dose before titration** | **0** |  | -0.7 (-0.8, -0.6) #m=471 | -1.0 (-1.1, -0.9) #m=1283 |  | -1.2 (-1.3, -1.0) #m=364 |  |  | -0.1 (-0.6, 0.3) #m=41 |  |  |  |
|  | **2.5** | 0.7 (0.7, 0.8) #m=596 |  | -0.5 (-0.5, -0.4) #m=660 |  | -0.8 (-1.3, -0.3) #m=51 |  |  |  |  |  |  |
|  | **5** | 0.9 (0.9, 1.0) #m=818 | 0.4 (0.3, 0.4) #m=815 |  | -0.4 (-0.6, -0.1) #m=82 | -0.3 (-0.3, -0.2) #m=1686 |  |  |  |  |  |  |
|  | **7.5** |  |  | 0.2 (0.0, 0.5) #m=59 |  | -0.0 (-0.2, 0.1) #m=157 |  | -0.0 (-0.1, 0.1) #m=133 |  |  |  |  |
|  | **10** | 0.9 (0.8, 1.1) #m=260 | 0.3 (0.1, 0.5) #m=32 | 0.3 (0.3, 0.3) #m=1042 | -0.0 (-0.1, 0.1) #m=267 |  |  | -0.2 (-0.2, -0.1) #m=966 | -0.2 (-0.3, -0.2) #m=753 |  |  |  |
|  | **12.5** |  |  |  |  |  |  |  |  | 0.0 (-0.2, 0.2) #m=57 |  |  |
|  | **15** |  |  | 0.1 (-0.1, 0.3) #m=30 | 0.1 (0.0, 0.3) #m=50 | 0.2 (0.1, 0.3) #m=376 |  |  | -0.1 (-0.1, -0.0) #m=852 |  | -0.1 (-0.2, 0.1) #m=294 |  |
|  | **20** | 0.9 (0.5, 1.2) #m=47 |  |  |  | 0.2 (0.2, 0.3) #m=514 | -0.1 (-0.2, 0.0) #m=145 | 0.0 (-0.0, 0.1) #m=362 |  | -0.2 (-0.3, -0.1) #m=617 | -0.1 (-0.2, -0.0) #m=465 |  |
|  | **25** |  |  |  |  |  |  | -0.0 (-0.2, 0.2) #m=72 | 0.1 (-0.1, 0.2) #m=95 |  | -0.1 (-0.2, -0.0) #m=444 |  |
|  | **30** | 0.6 (0.2, 0.9) #m=45 |  |  |  | 0.2 (-0.1, 0.4) #m=68 |  | 0.3 (0.2, 0.4) #m=259 | 0.3 (0.2, 0.4) #m=323 | 0.2 (-0.0, 0.3) #m=83 |  | -0.3 (-0.5, -0.0) #m=73 |
|  | **40** |  |  |  |  |  |  |  |  |  |  |  |

Change in HbA1c values after glipizide initiation, titration or discontinuation. The values above the diagonal represent the instances where the medication has been initiated or up-titrated, while the values below the diagonal represent instances where the medication has been down-titrated or discontinued. The values refer to the mean difference in HbA1c (MD) and 95% confidence intervals. MD below 0 indicate a lowering in HbA1c while those above 0 indicate an increase in HbA1c. #m refers to the number of HbA1c pairs for that dose titration.

Table S1c. Change in HbA1c values (with HbA1c denoted in % units) after gliclazide titration.

|  |  | **Gliclazide dose after titration** | | | | | | | | | |
| --- | --- | --- | --- | --- | --- | --- | --- | --- | --- | --- | --- |
|  |  | **0** | **30** | **40** | **60** | **80** | **90** | **120** | **160** | **240** | **320** |
| **Gliclazide dose before titration** | **0** |  | -0.9 (-1.2, -0.7) #m=57 | -1.1 (-1.3, -0.9) #m=89 |  | -1.1 (-1.3, -1.0) #m=174 |  |  |  |  |  |
|  | **30** | 0.7 (0.4, 0.9) #m=44 |  |  | -0.2 (-0.4, -0.0) #m=73 |  |  |  |  |  |  |
|  | **40** | 1.2 (0.9, 1.4) #m=108 |  |  |  | -0.5 (-0.6, -0.3) #m=109 |  |  |  |  |  |
|  | **60** |  | 0.4 (0.2, 0.5) #m=49 |  |  |  | -0.3 (-0.6, 0.1) #m=38 | -0.1 (-0.5, 0.3) #m=33 |  |  |  |
|  | **80** | 1.0 (0.8, 1.2) #m=91 |  | 0.3 (0.2, 0.4) #m=171 |  |  |  |  | -0.3 (-0.4, -0.2) #m=233 |  |  |
|  | **90** |  |  |  |  |  |  |  |  |  |  |
|  | **120** |  |  |  |  |  |  |  |  | -0.3 (-0.5, -0.1) #m=34 |  |
|  | **160** |  |  |  |  | 0.3 (0.2, 0.4) #m=172 |  | -0.1 (-0.4, 0.1) #m=59 |  | -0.3 (-0.5, -0.1) #m=113 | -0.4 (-0.6, -0.2) #m=112 |
|  | **240** |  |  |  |  |  |  |  | 0.1 (-0.2, 0.3) #m=50 |  | -0.2 (-0.4, 0.1) #m=77 |
|  | **320** |  |  |  |  |  |  |  | 0.2 (0.1, 0.4) #m=119 | 0.3 (-0.2, 0.7) #m=31 |  |

Change in HbA1c values after gliclazide initiation, titration or discontinuation. The values above the diagonal represent the instances where the medication has been initiated or up-titrated, while the values below the diagonal represent instances where the medication has been down-titrated or discontinued. The values refer to the mean difference in HbA1c (MD) and 95% confidence intervals. MD below 0 indicate a lowering in HbA1c while those above 0 indicate an increase in HbA1c. #m refers to the number of HbA1c pairs for that dose titration.

Table S1d. Change in HbA1c values (with HbA1c denoted in % units) after tolbutamide titration.

|  |  | **Tolbutamide dose after titration** | | | | | | | | |
| --- | --- | --- | --- | --- | --- | --- | --- | --- | --- | --- |
|  |  | **0** | **250** | **500** | **750** | **1000** | **1500** | **2000** | **2250** | **3000** |
| **Tolbutamide dose before titration** | **0** |  | -0.7 (-1.0, -0.5) #m=52 | -1.1 (-1.3, -0.8) #m=103 |  |  |  |  |  |  |
|  | **250** | 0.5 (0.3, 0.6) #m=90 |  | -0.5 (-0.7, -0.3) #m=92 |  |  |  |  |  |  |
|  | **500** | 0.6 (0.5, 0.7) #m=177 | 0.3 (0.2, 0.4) #m=243 |  | -0.3 (-0.6, 0.0) #m=44 | -0.5 (-0.6, -0.4) #m=203 |  |  |  |  |
|  | **750** |  |  | 0.1 (-0.1, 0.2) #m=89 |  | 0.1 (-0.3, 0.5) #m=37 | -0.5 (-0.7, -0.2) #m=52 |  |  |  |
|  | **1000** | 0.7 (0.3, 1.0) #m=45 |  | 0.4 (0.3, 0.4) #m=277 |  |  | -0.3 (-0.5, -0.2) #m=187 |  |  |  |
|  | **1500** |  |  |  | 0.3 (0.2, 0.5) #m=64 | 0.1 (0.0, 0.2) #m=190 |  | -0.1 (-0.4, 0.2) #m=42 | -0.5 (-0.7, -0.3) #m=96 |  |
|  | **2000** |  |  |  |  | 0.6 (0.3, 1.0) #m=50 |  |  |  | -0.6 (-1.0, -0.3) #m=42 |
|  | **2250** |  |  |  |  |  | 0.4 (0.3, 0.5) #m=95 |  |  | -0.4 (-0.6, -0.2) #m=62 |
|  | **3000** |  |  |  |  |  | 0.5 (0.2, 0.8) #m=43 | 0.3 (0.2, 0.5) #m=67 |  |  |

Change in HbA1c values after tolbutamide initiation, titration or discontinuation. The values above the diagonal represent the instances where the medication has been initiated or up-titrated, while the values below the diagonal represent instances where the medication has been down-titrated or discontinued. The values refer to the mean difference in HbA1c (MD) and 95% confidence intervals. MD below 0 indicate a lowering in HbA1c while those above 0 indicate an increase in HbA1c. #m refers to the number of HbA1c pairs for that dose titration.

Table S1e. Change in HbA1c values (with HbA1c denoted in % units) after sitagliptin titration.

|  |  | **Sitagliptin dose after titration** | | | | |
| --- | --- | --- | --- | --- | --- | --- |
|  |  | **0** | **25** | **50** | **75** | **100** |
| **Sitagliptin dose before titration** | **0** |  | -0.7 (-0.8, -0.5) #m=266 | -0.7 (-0.8, -0.6) #m=411 |  | -0.5 (-0.8, -0.2) #m=86 |
|  | **25** | 0.7 (0.5, 0.8) #m=93 |  | -0.3 (-0.4, -0.2) #m=225 |  |  |
|  | **50** | 0.7 (0.5, 0.9) #m=156 | 0.3 (0.0, 0.5) #m=56 |  | -0.3 (-0.6, -0.0) #m=43 | -0.2 (-0.4, -0.1) #m=439 |
|  | **75** |  |  |  |  | 0.1 (-0.3, 0.5) #m=35 |
|  | **100** | 0.6 (0.4, 0.8) #m=82 |  | 0.2 (0.0, 0.3) #m=91 |  |  |

Change in HbA1c values after sitagliptin initiation, titration or discontinuation. The values above the diagonal represent the instances where the medication has been initiated or up-titrated, while the values below the diagonal represent instances where the medication has been down-titrated or discontinued. The values refer to the mean difference in HbA1c (MD) and 95% confidence intervals. MD below 0 indicate a lowering in HbA1c while those above 0 indicate an increase in HbA1c. #m refers to the number of HbA1c pairs for that dose titration.

Table S1f. Change in HbA1c values (with HbA1c denoted in % units) after linaliptin titration.

|  |  | **Linagliptin dose after titration** | | |
| --- | --- | --- | --- | --- |
|  |  | **0** | **2.5** | **5** |
| **Linagliptin dose before titration** | **0** |  | -0.8 (-1.0, -0.6) #m=139 | -0.9 (-0.9, -0.8) #m=1807 |
|  | **2.5** | 0.5 (0.2, 0.8) #m=32 |  | -0.3 (-0.5, -0.1) #m=79 |
|  | **5** | 0.6 (0.5, 0.7) #m=363 | 0.2 (0.0, 0.4) #m=36 |  |

Change in HbA1c values after linagliptin initiation, titration or discontinuation. The values above the diagonal represent the instances where the medication has been initiated or up-titrated, while the values below the diagonal represent instances where the medication has been down-titrated or discontinued. The values refer to the mean difference in HbA1c (MD) and 95% confidence intervals. MD below 0 indicate a lowering in HbA1c while those above 0 indicate an increase in HbA1c. #m refers to the number of HbA1c pairs for that dose titration.

Table S1g. Change in HbA1c values (with HbA1c denoted in % units) after dapagliflozin titration.

|  |  | **Dapagliflozin dose after titration** | | | |
| --- | --- | --- | --- | --- | --- |
|  |  | **0** | **2.5** | **5** | **10** |
| **Dapagliflozin dose before titration** | **0** |  | -0.8 (-1.0, -0.5) #m=67 | -0.8 (-0.9, -0.7) #m=570 | -1.0 (-1.0, -0.9) #m=722 |
|  | **2.5** |  |  | -0.1 (-0.4, 0.2) #m=39 |  |
|  | **5** | 0.3 (0.1, 0.6) #m=85 |  |  | -0.3 (-0.4, -0.2) #m=264 |
|  | **10** | 0.6 (0.3, 0.8) #m=96 |  | 0.1 (-0.1, 0.3) #m=76 |  |

Change in HbA1c values after dapagliflozin initiation, titration or discontinuation. The values above the diagonal represent the instances where the medication has been initiated or up-titrated, while the values below the diagonal represent instances where the medication has been down-titrated or discontinued. The values refer to the mean difference in HbA1c (MD) and 95% confidence intervals. MD below 0 indicate a lowering in HbA1c while those above 0 indicate an increase in HbA1c. #m refers to the number of HbA1c pairs for that dose titration.

Table S1h. Change in HbA1c values (with HbA1c denoted in % units) after empagliflozin titration.

|  |  | **Empagliflozin dose after titration** | | |
| --- | --- | --- | --- | --- |
|  |  | **0** | **12.5** | **25** |
| **Empagliflozin dose before titration** | **0** |  | -0.7 (-0.9, -0.5) #m=152 | -0.6 (-0.8, -0.4) #m=101 |
|  | **12.5** |  |  |  |
|  | **25** |  |  |  |

Change in HbA1c values after empagliflozin initiation, titration or discontinuation. The values above the diagonal represent the instances where the medication has been initiated or up-titrated, while the values below the diagonal represent instances where the medication has been down-titrated or discontinued. The values refer to the mean difference in HbA1c (MD) and 95% confidence intervals. MD below 0 indicate a lowering in HbA1c while those above 0 indicate an increase in HbA1c. #m refers to the number of HbA1c pairs for that dose titration.

Table S1i. Change in HbA1c values (with HbA1c denoted in % units) after acarbose titration.

|  |  | **Acarbose dose after titration** | | | | | |
| --- | --- | --- | --- | --- | --- | --- | --- |
|  |  | **0** | **50** | **100** | **150** | **200** | **300** |
| **Acarbose dose before titration** | **0** |  | -0.3 (-0.5, -0.1) #m=49 | -0.4 (-0.6, -0.3) #m=221 | -0.6 (-0.7, -0.4) #m=211 |  | -0.6 (-1.0, -0.1) #m=37 |
|  | **50** | 0.2 (0.0, 0.4) #m=52 |  | -0.3 (-0.7, 0.0) #m=34 |  |  |  |
|  | **100** | 0.4 (0.2, 0.5) #m=149 | 0.3 (0.1, 0.5) #m=40 |  | -0.2 (-0.5, 0.0) #m=72 | -0.2 (-0.4, -0.0) #m=94 |  |
|  | **150** | 0.4 (0.2, 0.5) #m=122 |  | 0.0 (-0.1, 0.2) #m=65 |  |  | -0.2 (-0.3, 0.0) #m=144 |
|  | **200** | 0.4 (0.2, 0.6) #m=67 |  | 0.3 (0.2, 0.5) #m=48 |  |  | -0.1 (-0.3, 0.1) #m=75 |
|  | **300** | 0.6 (0.4, 0.9) #m=82 |  |  | 0.6 (0.3, 0.8) #m=49 | 0.3 (0.2, 0.5) #m=92 |  |

Change in HbA1c values after acarbose initiation, titration or discontinuation. The values above the diagonal represent the instances where the medication has been initiated or up-titrated, while the values below the diagonal represent instances where the medication has been down-titrated or discontinued. The values refer to the mean difference in HbA1c (MD) and 95% confidence intervals. MD below 0 indicate a lowering in HbA1c while those above 0 indicate an increase in HbA1c. #m refers to the number of HbA1c pairs for that dose titration
