# Supplementary material for: The effect of oral diabetes medications on glycated haemoglobin (HbA1c) in Asians in primary care: a retrospective cohort real-world data study
Source: BMC Med. 2022 Jan 26;20:22. doi: 10.1186/s12916-021-02221-z (PMC8790837; doi:10.1186/s12916-021-02221-z)
Supplement: Supplementary file 2 — Additional file 2. Additional file 2 of results from the analysis of “No OAD” and “Non-titrator” cohorts and multivariate analysis. Table S1, Figure S1, Table S2 and Table S3. Table S1. Change in HbA1c values (with HbA1c denoted in mmol/mol) in “No OAD” and “Non-titrators” cohorts. Figure S1. Visualization of covariates adjusted for in multivariate regression. Table S2. Multiple regression covariate weights (with HbA1c denoted in mmol/mol). Tables S3a to S3i. Change in HbA1c values (with HbA1c denoted in mmol/mol) after metformin titration (adjusted by regression). [file 12916_2021_2221_MOESM2_ESM.docx]

Table S1. Change in HbA1c values (with HbA1c denoted in mmol/mol) in “No OAD” and “Non-titrators” cohorts.

| **Oral anti-diabetic drug (OAD) combination** | **Mean change in HbA1c (mmol/mol)** | **95% CI** | **SD** | **Number of instances** |
| --- | --- | --- | --- | --- |
| **No OAD group** | | | | |
| No OAD | 0 | (0, 0) | 3 | 54744 |
| **Non-titrator group** | | | | |
| (Tolbutamide,250.0) | 0 | (-1, 0) | 6 | 300 |
| (Tolbutamide,500.0) | 0 | (-0, 1) | 7 | 365 |
| (Tolbutamide,750.0) | 0 | (-1, 1) | 4 | 54 |
| (Tolbutamide,1000.0) | 0 | (-1, 0) | 6 | 201 |
| (Tolbutamide,1500.0) | 0 | (-1, 1) | 6 | 94 |
| (Sitaglipitin,25.0) | 0 | (-2, 2) | 6 | 30 |
| (Sitaglipitin,50.0) | 0 | (-1, 1) | 4 | 54 |
| (Glipizide,2.5) | 0 | (-1, 0) | 7 | 469 |
| (Glipizide,5.0) | 0 | (0, 1) | 4 | 508 |
| (Glipizide,10.0) | 0 | (-1, 1) | 6 | 207 |
| (Glipizide,20.0) | -1 | (-2, 1) | 6 | 55 |
| (Acarbose,100.0) | 0 | (-1, 1) | 4 | 41 |
| (Gliclazide,30.0) | 0 | (-1, 1) | 4 | 56 |
| (Gliclazide,40.0) | 0 | (-1, 1) | 6 | 130 |
| (Gliclazide,80.0) | 0 | (-2, 1) | 7 | 51 |
| (Metformin,125.0) | 0 | (0, 0) | 3 | 385 |
| (Metformin,250.0) | 0 | (0, 0) | 4 | 3592 |
| (Metformin,250.0),(Glipizide,5.0) | 0 | (-2, 1) | 4 | 39 |
| (Metformin,500.0) | 0 | (0, 0) | 4 | 6472 |
| (Metformin,500.0),(Tolbutamide,500.0) | 0 | (-1, 1) | 4 | 105 |
| (Metformin,500.0),(Tolbutamide,1000.0) | 0 | (-1, 1) | 4 | 114 |
| (Metformin,500.0),(Glipizide,2.5) | 0 | (-1, 1) | 4 | 82 |
| (Metformin,500.0),(Glipizide,5.0) | -1 | (-1, 0) | 4 | 148 |
| (Metformin,500.0),(Glipizide,10.0) | 0 | (-1, 1) | 7 | 115 |
| (Metformin,500.0),(Gliclazide,30.0) | 0 | (-1, 2) | 3 | 33 |
| (Metformin,750.0) | 0 | (0, 0) | 3 | 235 |
| (Metformin,750.0),(Glipizide,5.0) | 0 | (-1, 2) | 4 | 38 |
| (Metformin,850.0) | 0 | (-1,1) | 4 | 64 |
| (Metformin,1000.0) | 0 | (0, 0) | 4 | 5745 |
| (Metformin,1000.0),(Tolbutamide,500.0) | 0 | (-1, 1) | 6 | 163 |
| (Metformin,1000.0),(Tolbutamide,1000.0) | 0 | (-1, 1) | 6 | 160 |
| (Metformin,1000.0),(Sitaglipitin,50.0) | 0 | (-1, 2) | 4 | 35 |
| (Metformin,1000.0),(Glipizide,2.5) | 0 | (-1, 1) | 4 | 187 |
| (Metformin,1000.0),(Glipizide,5.0) | 0 | (0, 1) | 6 | 555 |
| (Metformin,1000.0),(Glipizide,10.0) | 0 | (0, 1) | 7 | 482 |
| (Metformin,1000.0),(Glipizide,15.0) | -1 | (-2, 1) | 8 | 99 |
| (Metformin,1000.0),(Glipizide,20.0) | 0 | (-1, 1) | 4 | 72 |
| (Metformin,1000.0),(Gliclazide,80.0) | -1 | (-4, 2) | 14 | 56 |
| (Metformin,1000.0),(Gliclazide,160.0) | 0 | (-1, 1) | 6 | 53 |
| (Metformin,1000.0),(Gliclazide,320.0) | 0 | (-1, 1) | 4 | 38 |
| (Metformin,1500.0) | 0 | (0, 0) | 4 | 1520 |
| (Metformin,1500.0),(Tolbutamide,1000.0) | 0 | (-1, 2) | 4 | 36 |
| (Metformin,1500.0),(Tolbutamide,1500.0) | 0 | (-1, 1) | 6 | 126 |
| (Metformin,1500.0),(Glipizide,2.5) | 0 | (-1, 1) | 4 | 54 |
| (Metformin,1500.0),(Glipizide,5.0) | 0 | (-1, 1) | 7 | 216 |
| (Metformin,1500.0),(Glipizide,10.0) | 0 | (0, 1) | 6 | 301 |
| (Metformin,1500.0),(Glipizide,15.0) | 0 | (-2, 1) | 4 | 40 |
| (Metformin,1500.0),(Glipizide,20.0) | 1 | (-1, 2) | 6 | 55 |
| (Metformin,1700.0) | 0 | (0, 0) | 4 | 1655 |
| (Metformin,1700.0),(Tolbutamide,500.0) | 0 | (-2, 1) | 4 | 31 |
| (Metformin,1700.0),(Tolbutamide,1000.0) | 0 | (-1, 1) | 4 | 62 |
| (Metformin,1700.0),(Tolbutamide,1500.0) | 0 | (-1, 1) | 4 | 46 |
| (Metformin,1700.0),(Glipizide,2.5) | 0 | (-1, 1) | 4 | 48 |
| (Metformin,1700.0),(Glipizide,5.0) | 0 | (0, 1) | 6 | 305 |
| (Metformin,1700.0),(Glipizide,10.0) | 0 | (0, 1) | 6 | 379 |
| (Metformin,1700.0),(Glipizide,20.0) | -1 | (-1, 0) | 6 | 105 |
| (Metformin,1700.0),(Glipizide,30.0) | 1 | (0, 2) | 4 | 68 |
| (Metformin,1700.0),(Gliclazide,30.0) | 0 | (-2,2) | 4 | 31 |
| (Metformin,1700.0),(Gliclazide,80.0) | 0 | (-1, 1) | 4 | 68 |
| (Metformin,1700.0),(Gliclazide,160.0) | 0 | (-1, 1) | 4 | 62 |
| (Metformin,1700.0),(Gliclazide,320.0) | 0 | (-1, 2) | 6 | 30 |
| (Metformin,2000.0) | 0 | (-1, 1) | 6 | 166 |
| (Metformin,2000.0),(Glipizide,5.0) | 0 | (-1, 1) | 6 | 80 |
| (Metformin,2000.0),(Glipizide,10.0) | -1 | (-2, 1) | 7 | 73 |
| (Metformin,2000.0),(Glipizide,20.0) | 0 | (-1, 1) | 4 | 63 |
| (Metformin,2250.0) | 0 | (0, 0) | 4 | 294 |
| (Metformin,2250.0),(Glipizide,5.0) | 0 | (-1, 1) | 6 | 64 |
| (Metformin,2250.0),(Glipizide,10.0) | 0 | (-1, 1) | 7 | 61 |
| (Metformin,2550.0) | 0 | (0, 0) | 4 | 563 |
| (Metformin,2550.0),(Tolbutamide,1000.0) | -1 | (-2, 0) | 3 | 41 |
| (Metformin,2550.0),(Tolbutamide,1500.0) | 0 | (-2, 2) | 7 | 38 |
| (Metformin,2550.0),(Tolbutamide,3000.0) | 3 | (-1, 7) | 10 | 30 |
| (Metformin,2550.0),(Glipizide,5.0) | 0 | (-1, 1) | 4 | 112 |
| (Metformin,2550.0),(Glipizide,10.0) | 0 | (-1, 0) | 4 | 245 |
| (Metformin,2550.0),(Glipizide,15.0) | 0 | (-2, 2) | 7 | 37 |
| (Metformin,2550.0),(Glipizide,20.0) | 0 | (-1, 1) | 6 | 161 |
| (Metformin,2550.0),(Glipizide,30.0) | 0 | (-1, 1) | 7 | 97 |
| (Metformin,2550.0),(Glipizide,30.0),(Sitaglipitin,50.0) | 0 | (-2, 1) | 6 | 31 |
| (Metformin,2550.0),(Acarbose,300.0),(Glipizide,30.0) | 1 | (-1, 2) | 6 | 57 |
| (Metformin,2550.0),(Gliclazide,80.0) | 0 | (-3, 3) | 11 | 49 |
| (Metformin,2550.0),(Gliclazide,160.0) | 0 | (-1, 1) | 6 | 52 |
| (Metformin,2550.0),(Gliclazide,320.0) | 1 | (0, 3) | 6 | 48 |
| (Metformin,3000.0) | 0 | (-1, 1) | 6 | 107 |
| (Metformin,3000.0),(Tolbutamide,1500.0) | 1 | (-1, 3) | 7 | 46 |
| (Metformin,3000.0),(Tolbutamide,3000.0) | 1 | (-2, 3) | 7 | 35 |
| (Metformin,3000.0),(Glipizide,5.0) | 0 | (-1, 1) | 4 | 46 |
| (Metformin,3000.0),(Glipizide,30.0) | 1 | (-1, 4) | 9 | 32 |
| (Metformin,3000.0),(Glipizide,30.0),(Sitaglipitin,100.0) | 1 | (-1, 4) | 9 | 37 |
| (Metformin,3000.0),(Acarbose,150.0),(Glipizide,30.0) | 0 | (-2, 3) | 9 | 40 |
| (Metformin,3000.0),(Acarbose,300.0),(Glipizide,20.0) | 0 | (-1, 1) | 6 | 40 |
| (Metformin,3000.0),(Acarbose,300.0),(Glipizide,30.0) | 0 | (-1, 2) | 7 | 65 |
|  |  |  |  |  |

Change in HbA1c values between consecutive OAD prescriptions without any titration. The OAD combination refers to the combination of OAD in the consecutive prescriptions. The number accompanying the OAD name refers to the total daily dose of the OAD. Abbreviations: HbA1c = glycosylated hemoglobin, OAD = oral anti-diabetic drug, SD = standard deviation, 95% CI = 95% confidence interval.

Figure S1. Visualization of covariates adjusted for in multivariate regression.


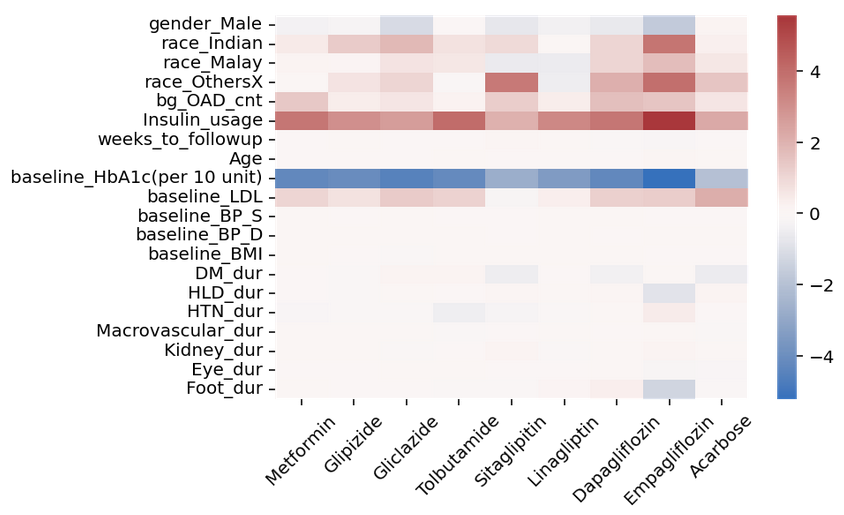


*With multivariate regression, the weights of the coviates shown in the figure were computed for each individual medication. The figure shows that baseline HbA1c and insulin use are the two covariates with the largest impact on HbA1c change. The unit of baseline HbA1c is in mmol/mol.*

Table S2. Multiple regression covariate weights (with HbA1c denoted in mmol/mol).

|  | **Metformin** | **Glipizide** | **Gliclazide** | **Tolbutamide** | **Sitaglipitin** | **Linagliptin** | **Dapagliflozin** | **Empagliflozin** | **Acarbose** |
| --- | --- | --- | --- | --- | --- | --- | --- | --- | --- |
| **gender_Male** | -0.3432 | -0.2571 | -1.1359 | -0.0576 | -0.7346 | -0.3974 | -0.6357 | -1.7039 | 0.2022 |
| **race_Indian** | 0.4894 | 1.3136 | 1.8273 | 0.6858 | 0.9432 | 0.0241 | 1.064 | 3.7802 | 0.3379 |
| **race_Malay** | 0.1912 | 0.1491 | 0.6464 | 0.5609 | -0.5958 | -0.5695 | 1.0773 | 1.7242 | 0.5708 |
| **race_OthersX** | 0.0521 | 0.6486 | 1.0661 | -0.124 | 3.6576 | -0.5108 | 2.0849 | 3.955 | 1.4787 |
| **bg_OAD_cnt** | 1.3943 | 0.4208 | 0.6055 | 0.2422 | 1.2995 | 0.3975 | 1.6519 | 1.4838 | 0.6165 |
| **Insulin_usage** | 3.7131 | 3.0139 | 2.6371 | 4.0637 | 2.0309 | 3.2058 | 3.7447 | 5.5603 | 2.2454 |
| **weeks_to_followup** | -0.0085 | 0.0367 | -0.0249 | -0.0182 | 0.0907 | 0.0068 | -0.1104 | -0.1676 | -0.0213 |
| **Age** | -0.0327 | -0.0462 | -0.0406 | 0.0296 | -0.0433 | -0.0685 | -0.0036 | 0.1229 | 0.0102 |
| **baseline_HbA1c(per 10 unit)** | -4.289 | -4.105 | -4.512 | -4.207 | -2.738 | -3.469 | -4.285 | -5.218 | -2.041 |
| **baseline_LDL** | 1.0883 | 0.7017 | 1.3173 | 1.1583 | -0.2171 | 0.3704 | 1.1905 | 1.2716 | 2.1413 |
| **baseline_BP_S** | 0.019 | 0.0023 | 0.0104 | -0.0016 | -0.0018 | 0.0076 | 0.0417 | -0.0067 | -0.0374 |
| **baseline_BP_D** | 0.0196 | 0.0121 | 0.0237 | 0.0089 | -0.0048 | 0.0089 | -0.066 | 0.0869 | 0.0329 |
| **baseline_BMI** | 0.0495 | -0.0015 | -0.0816 | 0.0758 | 0.0062 | 0.0838 | 0.0416 | -0.0049 | -0.0276 |
| **DM_dur** | -0.0396 | -0.0974 | 0.1735 | 0.174 | -0.5088 | -0.0038 | -0.3884 | 0.0155 | -0.5569 |
| **HLD_dur** | -0.0753 | -0.1068 | 0.0372 | -0.0463 | 0.0918 | 0.0082 | 0.1034 | -0.8808 | 0.2069 |
| **HTN_dur** | -0.1733 | -0.1369 | -0.1556 | -0.4638 | -0.2622 | -0.1494 | 0.0521 | 0.43 | -0.08 |
| **Macrovascular_dur** | 0.0077 | 0.0197 | 0.0382 | -0.1229 | -0.0131 | 0.0078 | -0.0492 | 0.0103 | -0.1106 |
| **Kidney_dur** | 0.0837 | 0.0683 | -0.1539 | -0.0024 | 0.1986 | -0.0972 | 0.0528 | 0.1828 | 0.0091 |
| **Eye_dur** | -0.0141 | -0.0352 | 0.0184 | 0.0335 | -0.0036 | -0.0693 | 0.0823 | -0.2497 | -0.1928 |
| **Foot_dur** | 0.0797 | -0.0419 | -0.0691 | -0.1615 | -0.155 | 0.1535 | 0.3512 | -1.3433 | -0.102 |

Table S3a. Change in HbA1c values (with HbA1c denoted in mmol/mol) after metformin titration (adjusted).

|  |  | **Metformin dose after titration** | | | | | | | | | | | | |
| --- | --- | --- | --- | --- | --- | --- | --- | --- | --- | --- | --- | --- | --- | --- |
|  |  | **0** | **125** | **250** | **500** | **750** | **850** | **1000** | **1500** | **1700** | **2000** | **2250** | **2550** | **3000** |
| **Metformin dose before titration** | **0** |  |  | -4 #m=487 | -6 #m=1849 |  |  | -8 #m=676 | -9 #m=36 | -10 #m=60 |  |  |  |  |
|  | **125** |  |  | -5 #m=34 |  |  |  |  |  |  |  |  |  |  |
|  | **250** | 1 #m=173 | -3 #m=105 |  | -4 #m=906 |  |  | -6 #m=87 |  |  |  |  |  |  |
|  | **500** | 2 #m=220 |  | -2 #m=1412 |  | -4 #m=282 |  | -6 #m=3108 | -7 #m=77 | -8 #m=53 |  |  |  |  |
|  | **750** |  |  |  | -1 #m=182 |  |  | -4 #m=225 | -5 #m=190 | -6 #m=31 |  |  |  |  |
|  | **850** |  |  |  | 0 #m=34 |  |  | -2 #m=58 |  | -4 #m=161 |  |  | -6 #m=39 |  |
|  | **1000** | 5 #m=137 |  | 1 #m=31 | -1 #m=1953 | -1 #m=105 | -3 #m=57 |  | -4 #m=2150 | -5 #m=1670 | -5 #m=198 |  | -7 #m=47 | -6 #m=47 |
|  | **1500** | 6 #m=41 |  |  | 1 #m=96 | 1 #m=91 |  | -1 #m=1128 |  | -3 #m=739 | -3 #m=418 | -4 #m=498 | -5 #m=398 |  |
|  | **1700** | 8 #m=46 |  |  | 2 #m=38 |  | 0 #m=240 | 0 #m=773 | -1 #m=142 |  | -2 #m=611 |  | -4 #m=1154 |  |
|  | **2000** |  |  |  |  |  |  | 1 #m=228 | 0 #m=82 | -1 #m=127 |  |  | -3 #m=179 | -2 #m=458 |
|  | **2250** |  |  |  |  |  |  | 1 #m=34 | 0 #m=227 | -2 #m=30 | -1 #m=42 |  | -3 #m=311 | -2 #m=124 |
|  | **2550** |  |  |  |  |  | 1 #m=30 | 2 #m=75 | 0 #m=165 | -1 #m=920 | 0 #m=182 | -2 #m=33 |  | -1 #m=642 |
|  | **3000** |  |  |  |  |  |  | 3 #m=41 | 2 #m=65 |  | 1 #m=438 | -1 #m=35 | -1 #m=129 |  |

Change in HbA1c values after metformin initiation, titration or discontinuation. The values above the diagonal represent the instances where the medication has been initiated or up-titrated, while the values below the diagonal represent instances where the medication has been down-titrated or discontinued. The values refer to the mean difference in HbA1c (MD) and 95% confidence intervals. MD below 0 indicate a lowering in HbA1c while those above 0 indicate an increase in HbA1c. #m refers to the number of HbA1c pairs for that dose titration.

Table S3b. Change in HbA1c values (with HbA1c denoted in mmol/mol) after glipizide titration (adjusted).

|  |  | **Glipizide dose after titration** | | | | | | | | | | |
| --- | --- | --- | --- | --- | --- | --- | --- | --- | --- | --- | --- | --- |
|  |  | **0** | **2.5** | **5** | **7.5** | **10** | **12.5** | **15** | **20** | **25** | **30** | **40** |
| **Glipizide dose before titration** | **0** |  | -8 #m=471 | -9 #m=1283 |  | -9 #m=364 |  |  | -7 #m=41 |  |  |  |
|  | **2.5** | 2 #m=596 |  | -5 #m=660 |  | -4 #m=51 |  |  |  |  |  |  |
|  | **5** | 4 #m=818 | -1 #m=815 |  | -2 #m=82 | -2 #m=1686 |  |  |  |  |  |  |
|  | **7.5** |  |  | -1 #m=59 |  | -1 #m=157 |  | 0 #m=133 |  |  |  |  |
|  | **10** | 5 #m=262 | -1 #m=32 | -2 #m=1042 | -1 #m=267 |  |  | 0 #m=966 | 0 #m=753 |  |  |  |
|  | **12.5** |  |  |  |  |  |  |  |  | 0 #m=57 |  |  |
|  | **15** |  |  | -2 #m=30 | -1 #m=50 | -1 #m=376 |  |  | 0 #m=852 |  | 2 #m=294 |  |
|  | **20** | 5 #m=47 |  |  |  | -2 #m=514 | 0 #m=145 | 0 #m=362 |  | 0 #m=617 | 1 #m=465 |  |
|  | **25** |  |  |  |  |  |  | -1 #m=72 | -1 #m=95 |  | 1 #m=444 |  |
|  | **30** | 5 #m=45 |  |  |  | -2 #m=68 |  | 0 #m=259 | 0 #m=323 | 0 #m=83 |  | 0 #m=73 |
|  | **40** |  |  |  |  |  |  |  |  |  |  |  |

Change in HbA1c values after glipizide initiation, titration or discontinuation. The values above the diagonal represent the instances where the medication has been initiated or up-titrated, while the values below the diagonal represent instances where the medication has been down-titrated or discontinued. The values refer to the mean difference in HbA1c (MD) and 95% confidence intervals. MD below 0 indicate a lowering in HbA1c while those above 0 indicate an increase in HbA1c. #m refers to the number of HbA1c pairs for that dose titration.

Table S3c. Change in HbA1c values (with HbA1c denoted in mmol/mol) after gliclazide titration (adjusted).

|  |  | **Gliclazide dose after titration** | | | | | | | | | |
| --- | --- | --- | --- | --- | --- | --- | --- | --- | --- | --- | --- |
|  |  | **0** | **30** | **40** | **60** | **80** | **90** | **120** | **160** | **240** | **320** |
| **Gliclazide dose before titration** | **0** |  | -8 #m=57 | -10 #m=89 |  | -9 #m=174 |  |  |  |  |  |
|  | **30** | 3 #m=44 |  |  | -2 #m=73 |  |  |  |  |  |  |
|  | **40** | 5 #m=108 |  |  |  | -3 #m=109 |  |  |  |  |  |
|  | **60** |  | -1 #m=49 |  |  |  | 1 #m=38 | 2 #m=33 |  |  |  |
|  | **80** | 6 #m=91 |  | -2 #m=171 |  |  |  |  | -1 #m=233 |  |  |
|  | **90** |  |  |  |  |  |  |  |  |  |  |
|  | **120** |  |  |  |  |  |  |  |  | 0 #m=34 |  |
|  | **160** |  |  |  |  | -2 #m=172 |  | 1 #m=59 |  | -1 #m=113 | 1 #m=112 |
|  | **240** |  |  |  |  |  |  |  | -1 #m=50 |  | 1 #m=77 |
|  | **320** |  |  |  |  |  |  |  | -1 #m=119 | -1 #m=31 |  |

Change in HbA1c values after gliclazide initiation, titration or discontinuation. The values above the diagonal represent the instances where the medication has been initiated or up-titrated, while the values below the diagonal represent instances where the medication has been down-titrated or discontinued. The values refer to the mean difference in HbA1c (MD) and 95% confidence intervals. MD below 0 indicate a lowering in HbA1c while those above 0 indicate an increase in HbA1c. #m refers to the number of HbA1c pairs for that dose titration.

Table S3d. Change in HbA1c values (with HbA1c denoted in mmol/mol) after tolbutamide titration (adjusted).

|  |  | **Tolbutamide dose after titration** | | | | | | | | |
| --- | --- | --- | --- | --- | --- | --- | --- | --- | --- | --- |
|  |  | **0** | **250** | **500** | **750** | **1000** | **1500** | **2000** | **2250** | **3000** |
| **Tolbutamide dose before titration** | **0** |  | -6 #m=52 | -8 #m=103 |  |  |  |  |  |  |
|  | **250** | 1 #m=90 |  | -4 #m=92 |  |  |  |  |  |  |
|  | **500** | 2 #m=177 | -2 #m=243 |  | -2 #m=44 | -3 #m=203 |  |  |  |  |
|  | **750** |  |  | -2 #m=89 |  | -2 #m=37 | -2 #m=52 |  |  |  |
|  | **1000** | 4 #m=45 |  | -1 #m=277 |  |  | -1 #m=187 |  |  |  |
|  | **1500** |  |  |  | 0 #m=64 | -2 #m=190 |  | -1 #m=42 | -3 #m=96 |  |
|  | **2000** |  |  |  |  | 0 #m=50 |  |  |  | 1 #m=42 |
|  | **2250** |  |  |  |  |  | -1 #m=95 |  |  | 0 #m=62 |
|  | **3000** |  |  |  |  |  | -1 #m=43 | 0 #m=67 |  |  |

Change in HbA1c values after tolbutamide initiation, titration or discontinuation. The values above the diagonal represent the instances where the medication has been initiated or up-titrated, while the values below the diagonal represent instances where the medication has been down-titrated or discontinued. The values refer to the mean difference in HbA1c (MD) and 95% confidence intervals. MD below 0 indicate a lowering in HbA1c while those above 0 indicate an increase in HbA1c. #m refers to the number of HbA1c pairs for that dose titration.

Table S3e. Change in HbA1c values (with HbA1c denoted in mmol/mol) after sitagliptin titration (adjusted).

|  |  | **Sitagliptin dose after titration** | | | | |
| --- | --- | --- | --- | --- | --- | --- |
|  |  | **0** | **25** | **50** | **75** | **100** |
| **Sitagliptin dose before titration** | **0** |  | -8 #m=266 | -8 #m=411 |  | -8 #m=86 |
|  | **25** | 2 #m=93 |  | -5 #m=225 |  |  |
|  | **50** | 3 #m=156 | -3 #m=56 |  | -4 #m=43 | -3 #m=439 |
|  | **75** |  |  |  |  | -1 #m=35 |
|  | **100** | 3 #m=82 |  | -4 #m=91 |  |  |

Change in HbA1c values after sitagliptin initiation, titration or discontinuation. The values above the diagonal represent the instances where the medication has been initiated or up-titrated, while the values below the diagonal represent instances where the medication has been down-titrated or discontinued. The values refer to the mean difference in HbA1c (MD) and 95% confidence intervals. MD below 0 indicate a lowering in HbA1c while those above 0 indicate an increase in HbA1c. #m refers to the number of HbA1c pairs for that dose titration.

Table S3f. Change in HbA1c values (with HbA1c denoted in mmol/mol) after linaliptin titration (adjusted).

|  |  | **Linagliptin dose after titration** | | |
| --- | --- | --- | --- | --- |
|  |  | **0** | **2.5** | **5** |
| **Linagliptin dose before titration** | **0** |  | -6 #m=139 | -7 #m=1807 |
|  | **2.5** | 4 #m=32 |  | -2 #m=79 |
|  | **5** | 4 #m=363 | -2 #m=36 |  |

Change in HbA1c values after linagliptin initiation, titration or discontinuation. The values above the diagonal represent the instances where the medication has been initiated or up-titrated, while the values below the diagonal represent instances where the medication has been down-titrated or discontinued. The values refer to the mean difference in HbA1c (MD) and 95% confidence intervals. MD below 0 indicate a lowering in HbA1c while those above 0 indicate an increase in HbA1c. #m refers to the number of HbA1c pairs for that dose titration.

Table S3g. Change in HbA1c values (with HbA1c denoted in mmol/mol) after dapagliflozin titration (adjusted).

|  |  | **Dapagliflozin dose after titration** | | | |
| --- | --- | --- | --- | --- | --- |
|  |  | **0** | **2.5** | **5** | **10** |
| **Dapagliflozin dose before titration** | **0** |  | -7 #m=67 | -8 #m=570 | -8 #m=722 |
|  | **2.5** |  |  | -3 #m=39 |  |
|  | **5** | 1 #m=85 |  |  | -4 #m=264 |
|  | **10** | 2 #m=96 |  | -2 #m=76 |  |

Change in HbA1c values after dapagliflozin initiation, titration or discontinuation. The values above the diagonal represent the instances where the medication has been initiated or up-titrated, while the values below the diagonal represent instances where the medication has been down-titrated or discontinued. The values refer to the mean difference in HbA1c (MD) and 95% confidence intervals. MD below 0 indicate a lowering in HbA1c while those above 0 indicate an increase in HbA1c. #m refers to the number of HbA1c pairs for that dose titration.

Table S3h. Change in HbA1c values (with HbA1c denoted in mmol/mol) after empagliflozin titration (adjusted).

|  |  | **Empagliflozin dose after titration** | | |
| --- | --- | --- | --- | --- |
|  |  | **0** | **12.5** | **25** |
| **Empagliflozin dose before titration** | **0** |  | -6 #m=152 | -3 #m=101 |
|  | **12.5** |  |  |  |
|  | **25** |  |  |  |

Change in HbA1c values after empagliflozin initiation, titration or discontinuation. The values above the diagonal represent the instances where the medication has been initiated or up-titrated, while the values below the diagonal represent instances where the medication has been down-titrated or discontinued. The values refer to the mean difference in HbA1c (MD) and 95% confidence intervals. MD below 0 indicate a lowering in HbA1c while those above 0 indicate an increase in HbA1c. #m refers to the number of HbA1c pairs for that dose titration.

Table S3i. Change in HbA1c values (with HbA1c denoted in mmol/mol) after acarbose titration (adjusted).

|  |  | **Acarbose dose after titration** | | | | | |
| --- | --- | --- | --- | --- | --- | --- | --- |
|  |  | **0** | **50** | **100** | **150** | **200** | **300** |
| **Acarbose dose before titration** | **0** |  | -3 #m=49 | -4 #m=221 | -5 #m=211 |  | -5 #m=37 |
|  | **50** | 0 #m=52 |  | -3 #m=34 |  |  |  |
|  | **100** | 1 #m=149 | 0 #m=40 |  | -2 #m=72 | -2 #m=94 |  |
|  | **150** | 2 #m=122 |  | -1 #m=65 |  |  | -1 #m=144 |
|  | **200** | 2 #m=67 |  | 0 #m=48 |  |  | -1 #m=75 |
|  | **300** | 5 #m=82 |  |  | 2 #m=49 | 2 #m=92 |  |

Change in HbA1c values after acarbose initiation, titration or discontinuation. The values above the diagonal represent the instances where the medication has been initiated or up-titrated, while the values below the diagonal represent instances where the medication has been down-titrated or discontinued. The values refer to the mean difference in HbA1c (MD) and 95% confidence intervals. MD below 0 indicate a lowering in HbA1c while those above 0 indicate an increase in HbA1c. #m refers to the number of HbA1c pairs for that dose titration.
